# Working memory gating across sensory modalities reveals modality-specific maintenance mechanisms

**DOI:** 10.1016/j.isci.2026.118017

**Published:** 2026-10-27

**Authors:** Ronja Limburg, Christian Beste, Astrid Prochnow

**Affiliations:** 1 Cognitive Neurophysiology, Department of Child and Adolescent Psychiatry, Faculty of Medicine, TU Dresden, 01307 Dresden, Saxony, Germany

**Keywords:** EEG, alpha band activity, beta band activity, audio-visual, beamforming

## Abstract

The flexible resolution of the conflict between updating and maintenance demands in working memory is thought to rely on a selective gating mechanism. While gating of visual information has been extensively studied, gating of multisensory information remains poorly understood. We addressed this gap by contrasting task performance and accompanying neuro-oscillatory modulations across a purely visual and two audiovisual variants of a working memory gating task. Task-dependent performance differences emerged specifically for gate closing. Across tasks, gate closing was consistently associated with alpha band activity modulations supporting the integration of task-relevant information and beta band activity modulations controlling the reconfiguration of the gate state, while theta band activity modulations were overall negligible. However, the functional localization and relative magnitude of these oscillatory modulations varied between tasks. Together, our findings suggest that working memory gating is not amodal, but that maintenance processes in particular are shaped by the sensory context.

## Highlights

• We compared working memory access control across sensory modalities
• Sensory format influenced working memory gate closing but not opening dynamics
• Neurophysiological analyses revealed distinct roles of alpha- and beta band activity

## Graphical abstract

**Figure:**
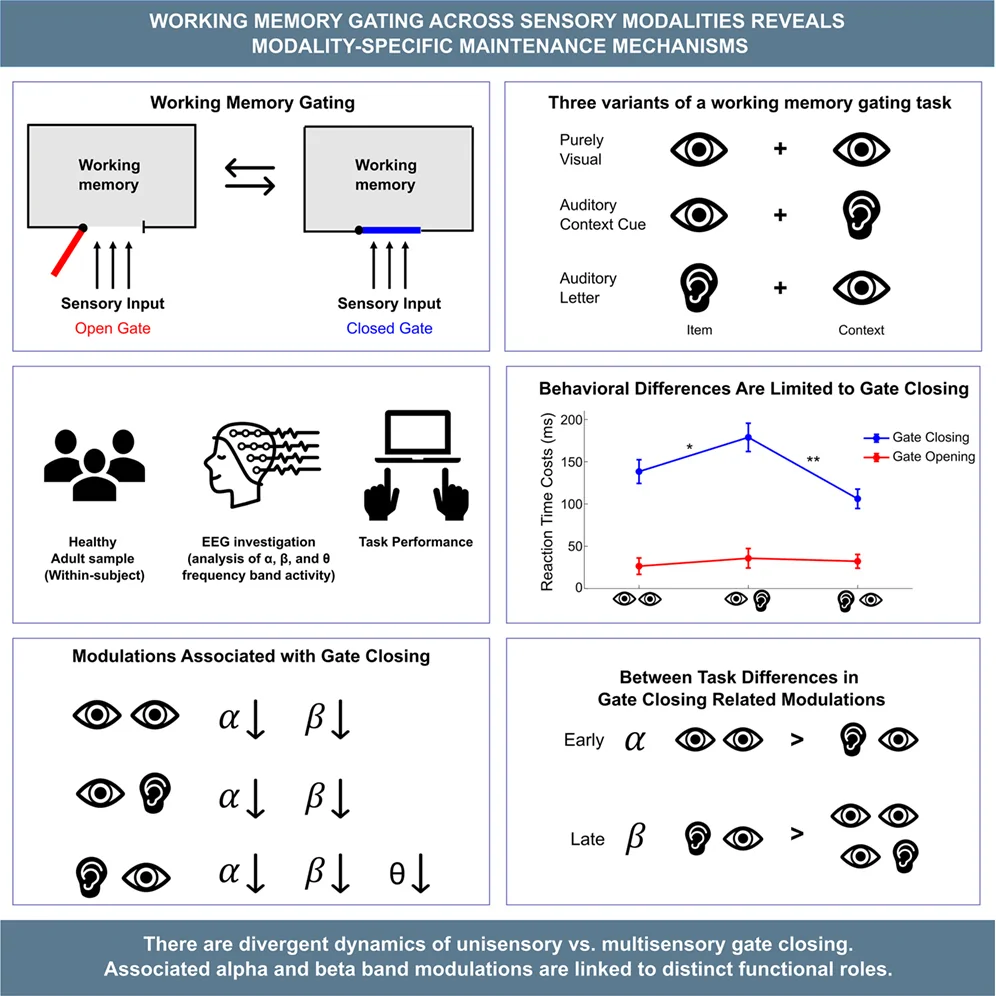

## Teaser

Systems neuroscience; Sensory neuroscience

## Introduction

Completing everyday activities such as conversing or following verbal instructions critically relies on two core functions of working memory (WM): The temporary storage of information and the manipulation and updating of stored information.^1^ Flexibly switching between these two complementary WM functions is considered to be a key component of cognitive control^2^ and is likely mediated by a gating mechanism that separates perceptual input from WM. When the gate is closed, maintenance of WM representations is supported by preventing interference from concurrent input. Conversely, opening the gate to WM permits updating of stored information.^3^^,^^4^^,^^5^ Gating and WM updating have traditionally been described as effortful processes that require a selective opening of the by-default closed WM gate,^3^^,^^6^ because switching from a closed to an opened gate or vice versa is accompanied by so-called switch costs, that is, increases in reaction times and error rates.^2^^,^^7^

Within this general conceptualization of a WM gating mechanism, an important, yet largely overlooked, consideration concerns the representational organization within WM. Influential models of WM architecture, such as Baddeley’s multicomponent model,^8^^,^^9^ highlight that information maintained in WM is inherently modality-specific, being distributed across subsystems specialized for visuospatial (visuospatial sketchpad) and auditory-verbal (phonological loop) information. Despite this distinction and recent theoretical advances emphasizing that gating not only concerns *when* information is updated but also *what* information is selected for updating and therefore granted access to WM,^6^ prior gating studies mainly focused on purely visual stimulus sets. As a consequence, it remains largely elusive how gating mechanisms operate when relevant information is presented across different sensory modalities, which would better reflect the demands of real-world cognition.^10^ A systematic examination of multisensory gating is therefore pivotal to provide a conceptual and empirical foundation for integrating the gating framework with modular accounts of WM architecture,^8^^,^^9^^,^^11^ two lines of theory that have developed in relative isolation.

In order to dissociate gate opening and gate closing, previous studies have routinely applied a variation of the n-back task referred to as the “Reference Back” paradigm.^12^^,^^13^ Within this paradigm, participants are shown a written letter and are instructed to compare the currently presented letter to the last reference letter maintained in WM. Reference trials are indicated by a certain frame color and require updating the stored reference, necessitating opening the gate to permit this updating process. On the other hand, comparison trials, indicated by a different frame color, require only a matching decision without updating WM contents; hence, the WM gate has to be closed to prevent updating of the reference letter. WM gating can thus be examined at the transitions between comparison and reference trials. Extending this paradigm beyond purely visual stimulus configurations to audiovisual stimuli could provide further insight into whether the WM gate should be understood as a single universal gate to working memory or whether, for instance, there may be different gates for different modalities. In the present study, we therefore employed three variants of the Reference Back task across which the sensory format of the item (i.e., letter) and the contextual cue (i.e., frame) were orthogonally manipulated. In addition to the original visual variant, we implemented an auditory context variant, in which the colored frames were replaced by pure tones of different frequencies, and an auditory letter variant, in which letters were presented verbally while the contextual cue remained visual.

Crucially, behavioral performance differences between purely visual and audiovisual task variants are not sufficient to inform about the processes that might differ depending on the modality: for instance, these differences could arise either from multisensory integration demands during perceptual processing or from modality-dependent modulations of gating-related control processes. The processing stage at which crossmodal influences on WM take place is still under debate.^10^ As behavioral data alone cannot disentangle these alternatives, we complemented the behavioral analysis with an investigation of neurophysiological activity modulations with high temporal precision (i.e., using EEG) during gate opening and closing. Previous studies have identified a consistent involvement of alpha, beta, and theta band activity (TBA) in gating dynamics.^14^^,^^15^^,^^16^^,^^17^ In particular, alpha band activity (ABA) decreases are evident during trials requiring a switch in gate state compared to trials in which the current gate state can be maintained. As ABA synchronization has been linked to attentional selection and selective inhibition,^18^^,^^19^^,^^20^^,^^21^ parietal ABA desynchronization has been interpreted as reflecting a transient broadening of information processing at critical decision points.^22^^,^^23^^,^^24^ Such a mechanism might be especially vital under multisensory conditions where attention has to be distributed across sensory modalities to allow the integration of item and contextual information. Accordingly, we expect stronger parietal ABA decreases in audiovisual compared to purely visual task variants. Moreover, beta band activity (BBA) synchronization has been linked to the maintenance of the current cognitive state and the preservation of the “status quo.”^25^^,^^26^^,^^27^^,^^28^ In line with this interpretation, previous work reported BBA decreases in fronto-parietal regions when a change in WM gate state is required, particularly during gate closing, reflecting the release of a stabilized cognitive configuration to enable a change in access control.^14^ Importantly, auditory information is often processed more rapidly and unfolds dynamically over time, which may induce a stronger tendency toward updating WM contents.^29^^,^^30^ Suppressing such an updating tendency during gate closing may therefore require enhanced stabilization of the current gate state, reflected in differential BBA modulations, particularly in audiovisual compared to purely visual task variants. Lastly, increased frontal TBA has been observed during gate opening and WM updating,^14^^,^^17^^,^^31^ but also during gate closing.^14^^,^^16^ This pattern suggests that TBA increases during WM gating might reflect a more general recruitment of cognitive control^32^ resulting from overall gate switching demands rather than a process uniquely tied to gate opening or closing.^16^ Notably, multisensory task variants may impose additional demands on such control processes.^29^^,^^30^ Consistent with this view, we assume enhanced frontal TBA during gate switching particularly in audiovisual task variants. Additionally, in order to clarify whether potential task differences in gating performance arise mainly from sensory-level modulations or whether they also implicate higher-order, supramodal control processes, we will complement the analysis of neuro-oscillatory modulations by applying a source estimation analysis. This approach will allow us to reconstruct the underlying generators of task-related differences in oscillatory activity and assess whether these effects localize to regions primarily implicated in sensory processing or might further extend into frontal association cortices linked to cognitive control processes.^32^

## Results

### Behavioral results

In the Reference Back task (Figure 1), the selective adaption of the WM gate state is captured by comparing performance (accuracy and reaction time [RT] measures) between trials requiring a gate state switch (trials in which the trial type changes relative to the preceding trial, that is, from reference to comparison or vice versa) in contrast to trials in which the gate state is constant relative to the preceding trial. Processes of gate opening, in particular, are captured through the contrast of switch/reference trials (trial 3 in Figure 1) and no-switch/reference trials (trial 4 in Figure 1), whereas gate closing processes are defined by the contrast between switch/comparison trials (trial 2 or 5 in Figure 1) and no-switch/comparison trials (trial 6 in Figure 1). Accordingly, gate opening and gate closing dynamics are reflected in the interaction between the task factors *trial type* and *switching*. As we wanted to assess modality-specific effects on WM gating, we compared gate opening and gate closing performance across our three task variants. Consequently, potential task-dependent differences would be reflected in a significant three-way interaction of the factors *task variant∗trial type∗switching*. Interpretation of this interaction can then be facilitated by expressing performance in terms of switch costs, rather than absolute accuracy and RT measures. Switch costs provide a measure of the *trial type∗switching* interaction by directly quantifying the gate opening and gate closing contrasts described above. Specifically, they are calculated as the performance difference between switch and corresponding no-switch conditions. Switch costs for reference trials then directly index the magnitude of gate opening costs, whereas switch costs for comparison trials signify the magnitude of gate closing costs. Given our assumption that auditory information might trigger a greater updating tendency that also takes more effort to be overridden in gate closing trials, we expected to observe lower gate opening but higher gate closing switch costs in the audiovisual compared to the purely visual task variant.

**Figure 1:**
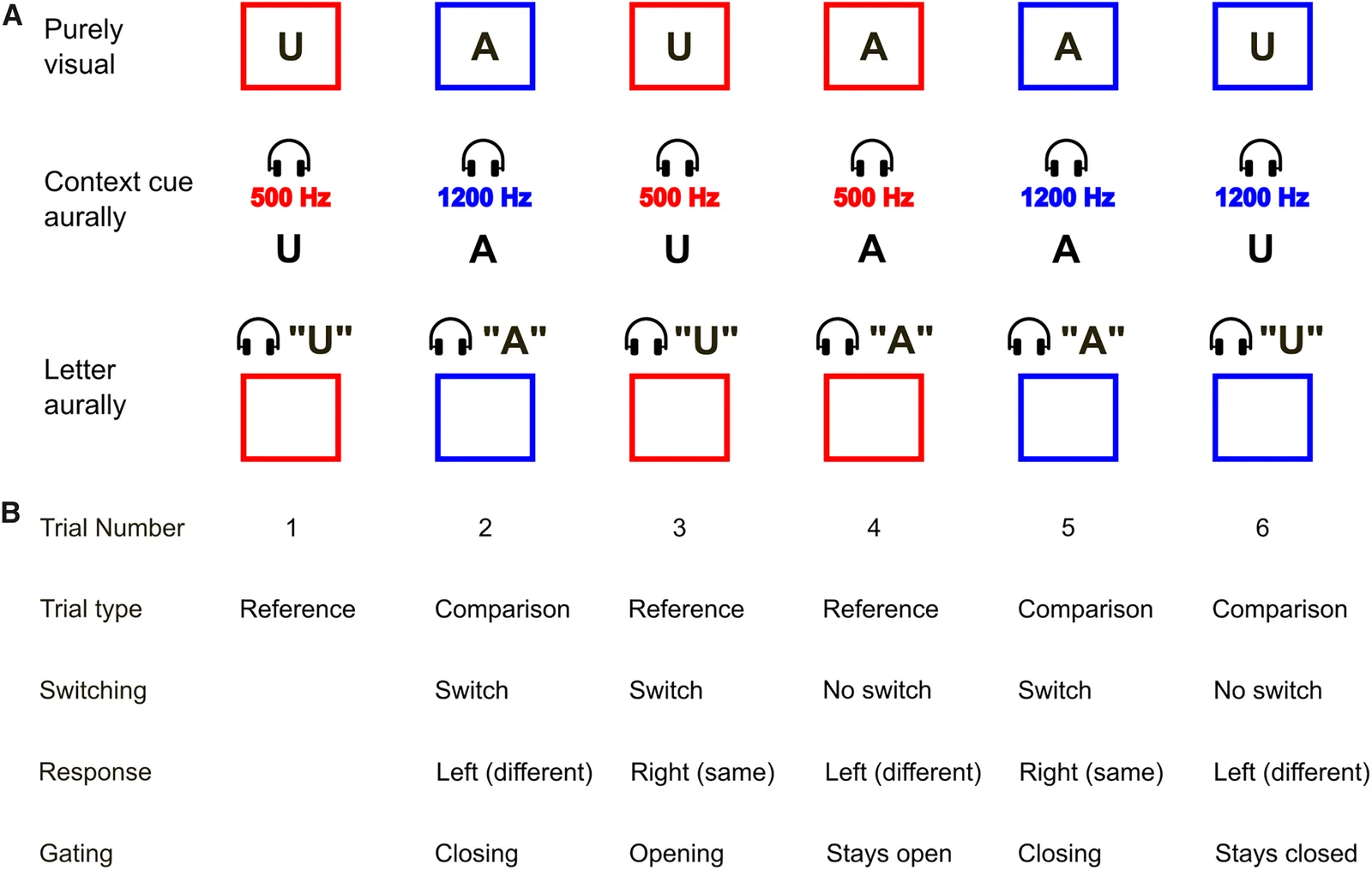
Illustration of the reference back task variants In figure part (A), an exemplary trial sequence and its differential implementation in the three task variants is illustrated. For audiovisual tasks, the headphone icon indicates which stimulus feature was presented in the auditory domain. Successive trials are displayed from left to right. Each task starts with a reference trial that does not require a response. Figure part (B) illustrates how each trial relates to central concepts in the Reference Back task.

Descriptive statistics for accuracy data can be found in Table 1. Behavioral trial rejection rates per condition in each condition can be found in Table 2. A repeated-measures ANOVA evaluating the influence of the within-subject factors *task variant* (1 = purely visual, 2 = context cue aurally, 3 = letter aurally), *trial type* (1 = reference trial, 2 = comparison trail), and *switching* (1 = switch, 2 = no-switch) on accuracy revealed a significant main effect of *task variant* (*F*(1.90, 62.72) = 9.75, *p* < 0.001, η^2^_*p*_ = 0.05), *trial type* (*F*(1, 33) = 13.14, *p* = 0.001, η^2^_*p*_ < 0.01), and *switching* (*F*(1, 33) = 4.74, *p* = 0.037, η^2^_*p*_ < 0.01). Moreover, the two-way interaction of *task variant∗trial type* was significant (*F*(1.84, 60.81)= 9.25, *p* < 0.001, η^2^_*p*_ < 0.01), as well as the interaction of *trial type∗switching* (*F*(1, 33)= 6.48, *p* = 0.016, η^2^_*p*_ < 0.01). Contrary, the interaction of *task variant∗switching (F*(1.76, 58.02) = 0.25, *p* = 0.751) and the three-way interaction of *task variant∗trial type∗switching* were not significant (*F*(1.86, 61.43) = 0.41, *p* = 0.649, BF = 0.11 ± 6.75%).

**Table 1:** Accuracy values observed in the three Reference Back variants

	purely visual	Context cue aurally	Letter aurally
ACC switch trials	92.0 (6.1)	89.3 (7.0)	92.2 (6.3)
ACC no-switch trials	92.9 (5.0)	89.9 (7.6)	92.9 (5.4)
ACC reference trials	91.5 (6.0)	89.8 (7.3)	91.8 (6.5)
ACC comparison trials	93.4 (5.0)	89.4 (7.4)	93.2 (5.1)
ACC gate opening	−4.2 (9.3)	−4.2 (10.4)	−3.2 (9.9)
ACC gate closing	0.5 (6.0)	2.0 (9.1)	0.5 (7.9)
ACC diff	−4.6 (12.7)	−6.1 (14.8)	−3.7 (15.5)

ACC = accuracy in percent, ACC gate opening = accuracy switch costs defined by the contrast between switch/reference minus no-switch/reference trials, ACC gate closing = accuracy switch costs defined by the contrast between switch/reference minus no-switch/reference trials, ACC diff = accuracy switch costs in gate opening minus accuracy switch costs in gate closing. Given are the means with standard deviations in parentheses.

**Table 2:** Behavioral trial rejection rates for the conditions used to evaluate gating dynamics in the three Reference Back variants

	purely visual	Context cue aurally	Letter aurally
switch/reference	9.3 (7.1)	11.2 (7.6)	11.6 (15.9)
no-switch/reference	7.2 (4.8)	9.1 (6.9)	10.1 (15.1)
switch/comparison	6.2 (4.7)	10.1 (6.4)	9.0 (15.0)
no-switch/comparison	6.4 (5.6)	11.1 (8.3)	9.4 (15.0)

Rejection rates in %. Rejection rates were calculated by dividing the number of retained trials with the number of trials per condition and subtracting this difference from 1. Given are the means with standard deviations in parentheses.

To resolve the interaction of *task variant∗trial type*, we applied Bonferroni-corrected *t*-tests, demonstrating that the accuracy difference between reference and comparison trials was only significant in the purely visual task (*t*(33) = −4.84, *p*_*corr*_ < 0.001, *d* = −0.37) and the auditory letter task (*t*(33) = −2.68, *p*_*corr*_ = 0.036, *d* = −0.25) but not in the auditory context cue task (*t*(33) = 1.32, *p*_*corr*_ = 0.585). Overall, accuracy in reference trials did not differ between task variants (purely visual versus auditory context cue task: *t*(33) = 1.96, *p*_*corr*_ = 0.349; purely visual versus auditory letter task: *t*(33) = −0.43, *p*_*corr*_ = 1; auditory context cue versus auditory letter task: *t*(33) = −2.30, *p*_*corr*_ = 0.169). Accuracy in comparison trials was similar between the purely visual and auditory letter tasks (*t*(33) = 0.27, *p*_*corr*_ = 1), but significantly reduced in the auditory context cue task compared to the purely visual task (*t*(33) = −4.70, *p*_*corr*_ < 0.001, *d* = −0.66) and the auditory letter task (*t*(33) = −5.01, *p*_*corr*_ < 0.001, *d* = −0.64).

Further inspection of the *trial type∗switching* interaction revealed that accuracy was lower during switch/reference than switch/comparison trials (*t*(33) = −3.61, *p*_*corr*_ = 0.002, *d* = −0.39), while similar accuracy was observed in no-switch/reference and no-switch/comparison trials (*t*(33) = 0.55, *p*_*corr*_ = 1). Furthermore, lower accuracy in switch compared to no-switch trials was only evident in reference trials (*t*(33) = −3.00, *p*_*corr*_ = 0.010, *d* = −0.34), but not comparison trials (*t*(33) = 0.96, *p*_*corr*_ = 0.691).

Despite the absence of the three-way interaction of *task variant∗trial type∗switching* that would have indicated task differences in gating performance, we further looked into the switch costs measure that directly reflects gate opening and gate closing costs. To this end, we conducted a repeated-measures ANOVA evaluating the influence of *task variant* (1 = purely visual, 2 = context cue aurally, 3 = letter aurally) and *gate switch mode* (1 = gate opening, 2 = gate closing) on accuracy switch costs. This analysis revealed a significant main effect of *gate switch mode* (*F*(1,33) = 6.48, *p* = 0.016, η^2^_*p*_ = 0.07), with accuracy reduction in gate opening being more pronounced (*M* = −3.8; *SD* = 9.8) than during gate closing (*M* = 1.0; *SD* = 7.7), see Figure 2A. In contrast, accuracy switch costs were not determined by the *task variant* (*F*(1.76,58.02) = 0.25, *p* = 0.751) or the *task variant∗gate switch mode* interaction (*F*(1.86,61.43) = 0.41, *p* = 0.649). The evaluation of accuracy switch cost differences between gate opening and closing also revealed no effects of *task variant* (*F*(2,66) = 0.41, *p* = 0.664).

**Figure 2:**
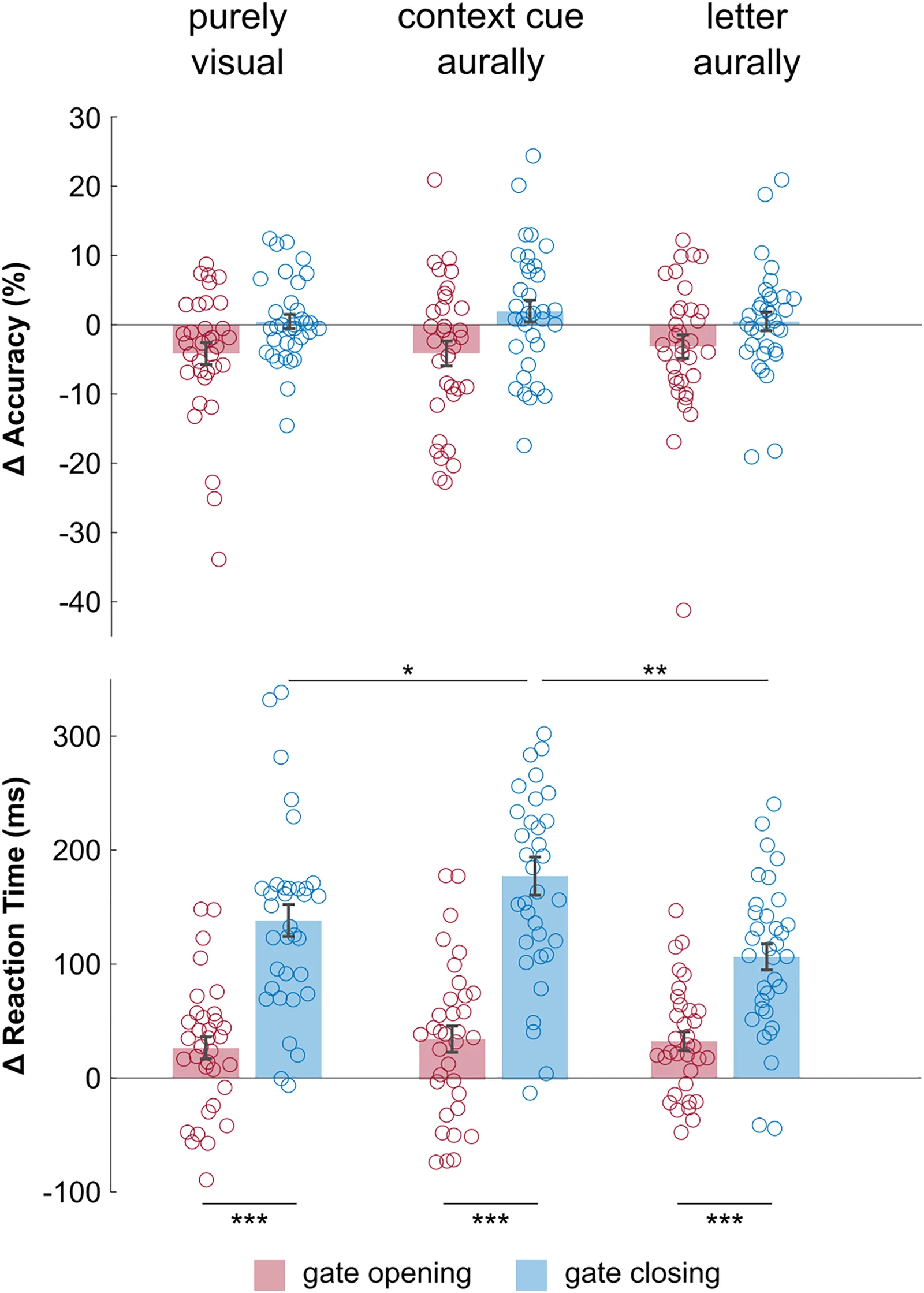
Behavioral results Displayed are switch costs as behavioral indicators of gating performance. Switch costs are changes in performance indicators associated with switching to a gate state in comparison to maintaining a certain gate state. In each column, accuracy-based switch costs (figure part A) and reaction time-based switch costs (figure part B) are displayed for a different task variant. Red bars indicate mean gate opening switch costs, while blue bars denote mean gate closing switch costs. Error bars represent the standard error of the mean. Circles depict individual data points for all subjects (∗*p* < 0.050, ∗∗*p* < 0.010, ∗∗∗*p* < 0.001; based on pairwise *t*-tests).

Overall, the accuracy results revealed comparable performance across tasks in reference trials. In contrast, accuracy in comparison trials was significantly lower in the auditory context cue task relative to the purely visual and the auditory letter task, suggesting that maintaining WM contents was particularly difficult in the auditory context cue task. However, we did not observe any task-specific differences in WM gating performance for neither the gate opening nor gate closing switch costs.

The descriptive statistics for the RTs can be found in Table 3. In the repeated-measures ANOVA evaluating how RTs are determined by the *task variant* (1 = purely visual, 2 = context cue aurally, 3 = letter aurally), *trial type* (1 = reference trial, 2 = comparison trial), and *switching* (1 = switch, 2 = no-switch), we observed a main effect of *task variant* (*F*(1.89, 62.36) = 250.5, *p* < 0.001, η^2^_*p*_ = 0.72), *trial type* (*F*(1, 33) = 77.8, *p* < 0.001, η^2^_*p*_ = 0.01), and *switching* (*F*(1.00, 33.00) = 213.9, *p* < 0.001, η^2^_*p*_ = 0.05). A significant *task variant∗trial type* interaction was not evident (*F*(1.76, 58.08) = 1.54, *p* = 0.225). Yet, the main effects were qualified by significant two-way interactions between *task variant∗switching* (*F*(1.54, 50.74) = 6.0, *p* = 0.008, η^2^_*p*_ < 0.01) and between *trial type∗switching* (*F*(1.00, 33.00) = 72.2, *p* < 0.001, η^2^_*p*_ = 0.02), as well as, importantly, a three-way interaction of *task variant∗trial type∗switching* (*F*(1.69, 55.78) = 6.2, *p* = 0.006, η^2^_*p*_ < 0.01). To unravel the three-way interaction, we subsequently computed a repeated-measures ANOVA in which we used RT switch costs instead of pure RTs as the dependent variable, as the RT switch costs capture the *trial type∗switching* interaction. Within this follow-up analysis, we probed how the *task variant* and *gate switch mode* (1 = gate opening, 2 = gate closing) affect RT switch costs. The analysis revealed significant main effect of *task variant* (*F*(1.54, 50.74) = 6.03, *p* = 0.008, η^2^_*p*_ = 0.05) and *gate switch mode* (*F*(1,33) = 72.20, *p* < 0.001, η^2^_*p*_ = 0.38). However, the main effects were qualified by an interaction of *task variant∗gate switch mode* (*F*(1.69,55.78) = 6.17, *p* = 0.006, η^2^_*p*_ = 0.04).

**Table 3:** Reaction times observed in the three Reference Back variants

	purely visual	Context cue aurally	Letter aurally
RT switch trials	564.8 (85.6)	568.8 (128.8)	865.7 (69.1)
RT no-switch trials	523.6 (76.6)	515.1 (109.0)	831.1 (72.2)
RT reference trials	552.3 (81.7)	554.6 (123.4)	860.1 (69.3)
RT comparison trials	536.1 (85.1)	529.2 (120.0)	836.8 (74.3)
RT gate opening	26.4 (56.5)	35.8 (67.3)	32.2 (47.5)
RT gate closing	138.0 (81.8)	179.0 (97.6)	106.0 (66.8)
RT diff	−112.0 (113.0)	−143.0 (93.1)	−74.0 (93.6)

RT = reaction time switch costs in ms, RT gate opening = reaction time switch costs defined by the contrast between switch/reference minus no-switch/reference trials, RT gate closing = reaction time switch costs defined by the contrast between switch/reference minus no-switch/reference trials, RT diff = reaction time switch costs in gate opening minus reaction time switch costs in gate closing. Given are the means with standard deviations in parentheses.

To examine this interaction more closely, we ran paired t-tests comparing gate opening and gate closing within each task variant. These tests revealed a significant gate opening versus gate closing difference in all task variants (purely visual: *t*(33) = −5.78, *p*_*corr*_ < 0.001, *d* = −1.59; context cue aurally: *t*(33) = −8.96, *p*_*corr*_ < 0.001, *d* = −1.71; letter aurally: *t*(33) = −4.61, *p*_*corr*_ < 0.001, *d* = −1.28). As depicted in Figure 2B, RT switch costs were always lower during gate opening than gate closing. Evaluating task differences for each gate switch mode separately using post-hoc repeated-measures ANOVAs, we observed no task effects on the magnitude of RT switch costs during gate opening (*F*(1.62,53.58) = 0.23, *p*_*corr*_ = 1, BF = 0.11 ± 0.57%). Yet, RT switch costs during gate closing were task-dependent (*F*(1.63,53.90) =10.57, *p*_*corr*_ < 0.001, η^2^_*p*_ = 0.12). Bonferroni-corrected pairwise comparisons revealed gate closing switch costs in the task variant presenting the context cue aurally to be higher than in the purely visual task variant (*t*(33) = 2.92, *p*_*corr*_ = 0.019, *d* = 0.25), and the task variant presenting the letter aurally (*t*(33) = 3.78, *p*_*corr*_ = 0.002, *d* = 0.42), whereas there was no difference between the purely visual task variant and the task variant presenting the letter aurally (*t*(33) = 2.33, *p*_*corr*_ = 0.077). The post-hoc repeated-measures ANOVA probing whether tasks differed in the magnitude of switch cost differences between gate switch modes revealed a significant main effect (*F*(1.69,55.78) = 6.17, *p* = 0.006, η^2^_*p*_ = 0.08). Pairwise comparisons with Bonferroni-adjusted *p*-values indicated that switch cost differences between gate switch modes were greater in the task variant presenting the context cue aurally than the task variant presenting the letter aurally (*t*(33) = −2.95, *p*_*corr*_ = 0.017, *d* = −0.74), while there were no differences between the purely visual task variant and the task variant presenting the context cue aurally (*t*(33) = 1.89, *p*_*corr*_ = 0.204), or the purely visual task variant and the task variant presenting the letter aurally (*t*(33) = −2.05, *p*_*corr*_ = 0.145).

In summary, the analysis of RT measures revealed that in all tasks, gate opening was associated with fewer RT costs than gate closing. While gate opening performance was comparable across tasks, gate closing performance was worse in the auditory context cue task, whereas the purely visual and auditory letter tasks did not differ from each other.

### Behavioral analysis including outliers

To ascertain the robustness of the reported behavioral results, we additionally performed the analyses outlined above on a sample that included RT outliers and exclusions due to poor EEG data quality.

To ensure that our definition of exclusion criteria did not inadvertently affect the pattern of results, we also performed the behavioral analysis under inclusion of the three participants deemed as reaction time outliers and the one participant excluded due to noisy EEG data. For the resulting sample of *N* = 38 participants, the repeated measures ANOVA probing the influence of the within-subject factors *task variant* (1 = purely visual, 2 = context cue aurally, 3 = letter aurally), *trial type* (1 = reference trial, 2 = comparison trial), and *switching* (1 = switch, 2 = no-switch) on accuracy suggested a main effect of *task variant* (*F*(1.99, 73.50) = 9.33, *p* < 0.001, η^2^_*p*_ = 0.03), *trial type* (*F*(1, 37) = 13.47, *p* < 0.001, η^2^_*p*_ < 0.01), and *switching* (*F*(1, 37) = 8.78, *p* = 0.005, η^2^_*p*_ < 0.01). In addition, the two-way interactions *task variant∗trial type (F*(1.85, 68.50) = 9.29, *p* < 0.001, η^2^_*p*_ < 0.01*)* and of *trial type∗switching* were significant (*F*(1, 37) = 9.03, *p* = 0.005, η^2^_*p*_ = 0.01), whereas the two-way interactions of *task variant∗switching (F*(1.77, 65.39) = 0.24, *p* = 0.760*)* and the three-way interaction *of task variant∗trial type∗switching (F*(1.89, 70.00) = 0.78, *p* = 0.458, BF = 0.11 ± 9.95%*)* were not significant.

To specify the interaction effect of *task variant∗trial type*, we applied Bonferroni-corrected t-tests evaluating whether the accuracy difference between reference and comparison trials is evident in all task variants. This approach revealed a significant distinction between trial types to be evident in the purely visual task (*t*(37) = −4.30, *p*_*corr*_ < 0.001, *d* = −0.31) and the auditory item task (*t*(37) = −3.39, *p*_*corr*_ = 0.005, *d* = −0.26), but not the auditory context cue task (*t*(37) = 1.10, *p*_*corr*_ = 0.833). There were no between-task differences in terms of accuracy during reference trials (purely visual versus auditory context cue task: *t*(37) = 2.44, *p*_*corr*_ = 0.118; purely visual versus auditory item task: *t*(37) = 0.56, *p*_*corr*_ = 1; auditory context cue versus auditory item task: *t*(37) = −1.63, *p*_*corr*_ = 674). Accuracy in comparison trials differed between the purely visual and auditory context cue task (*t*(37) = 4.92, *p*_*corr*_ < 0.001, *d* = 0.60) and between the auditory context cue task and the auditory item task (*t*(37) = −4.92, *p*_*corr*_ < 0.001, *d* = −0.51), but not between the purely visual and the auditory item task (*t*(37) = 0.61, *p*_*corr*_ = 1).

Closer examination of the *trial type∗switching* interaction revealed that accuracy differences between reference and comparison trials are evident during switch trials (*t*(37) = −4.20, *p*_*corr*_ < 0.001, *d* = −0.38), but not during no-switch trials (*t*(37) = 0.72, *p*_*corr*_ = 0.954). In reference trials, switching is accompanied by greater accuracy decreases than not switching (*t*(37) = −3.73, *p*_*corr*_ = 0.001, *d* = −0.37), whereas in comparison trials accuracy did not differ between switch and no-switch trials (*t*(37) = 0.87, *p*_*corr*_ = 0.776).

In line with our conceptual focus on gating performance, we further performed the repeated measures ANOVA with accuracy switch costs (see Figure 3 part A) as the dependent variable and *task variant* (1 = purely visual, 2 = context cue aurally, 3 = letter aurally) and *gate switch mode* (1 = gate opening, 2 = gate closing) as the within-subject predictors. It suggested that accuracy switch costs are significantly influenced by the *gate switch mode* (*F*(1, 37) = 9.03, *p* = 0.005, η^2^_*p*_ = 0.09), with higher accuracy switch costs in gate opening (*M* = −4.69; *SD* = 10.15) compared to gate closing trials (*M* = 0.86; *SD* = 7.99). whereas neither the *task variant* (*F*(1.77, 65.39) = 0.24, *p* = 0.760) nor the *task variant∗gate state* interaction (*F*(1.89, 70.00) = 0.78, *p* = 0.458) had a significant effect on accuracy switch costs.

**Figure 3:**
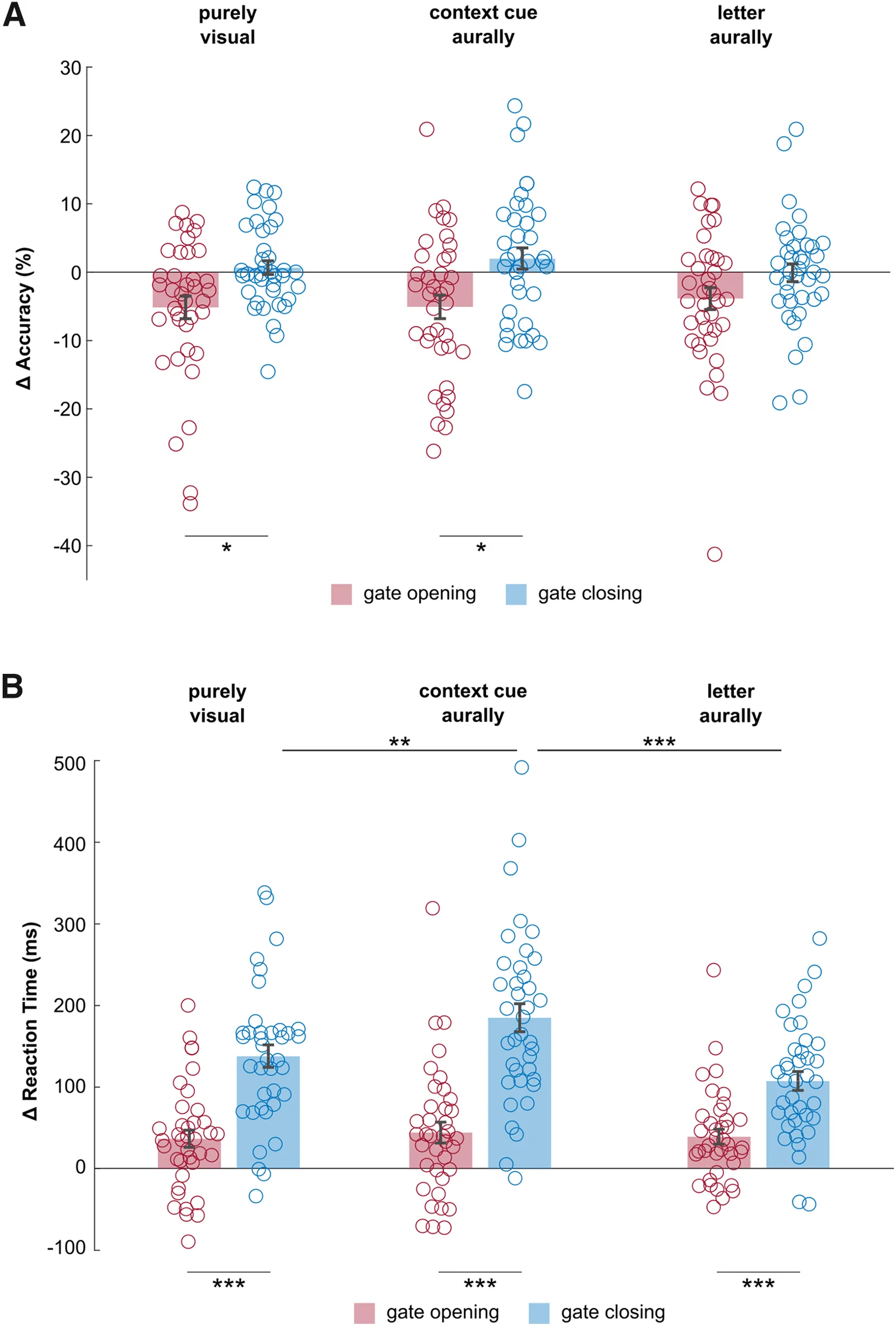
Behavioral results under consideration of outliers Displayed are switch costs as behavioral indicators of gating performance. Each column displays switch costs for a different task variant, with accuracy-based switch costs being illustrated in panel A and reaction time-based switch costs in panel B. Mean gate opening and gate closing switch costs are represented by red and blue bars, respectively, with error bars indicating the standard error of the mean and individual data points being represented by circles (∗*p* < 0.050, ∗∗*p* < 0.010, ∗∗∗*p* < 0.001; based on pairwise *t* tests).

Analysis of the factors influencing reaction times (RT) through a repeated measures ANOVA including the factors *task variant* (1 = purely visual, 2 = context cue aurally, 3 = letter aurally), *trial type* (1 = reference trial, 2 = comparison trial), and *switching* (1 = switch, 2 = no-switch) pointed at a significant main effect of the *task variant* (*F*(1.92, 71.12) = 290.71, *p* < 0.001, η^2^_*p*_ = 0.73), *trial type* (*F*(1, 37) = 77.18, *p* < 0.001, η^2^_*p*_ = 0.02), and *switching* (*F*(1, 37) = 185.08, *p* < 0.005, η^2^_*p*_ = 0.06). However, the main effects were qualified by a significant two-way interaction of *task variant∗switching* (*F*(1.58, 58.53) = 6.61, *p* = 0.005, η^2^_*p*_ < 0.01) and of *trial type∗switching* (*F*(1, 37) = 64.96, *p* < 0.001, η^2^_*p*_ = 0.02) as well as the three-way interaction of *task variant∗trial type∗switching* (*F*(1.80, 66.57) = 7.40, *p* = 0.002, η^2^_*p*_ < 0.01). The two-way interaction of *task variant∗trial type* (*F*(1.86, 68.70) = 1.19, *p* = 0.310).

To resolve the three-way interaction of *task variant∗trial type∗switching*, we performed a repeated measures ANOVA with the reaction time switch costs—which capture the trial type∗switching interaction—as the dependent variable and *task variant* (1 = purely visual, 2 = context cue aurally, 3 = letter aurally) and *gate switch mode* (1 = gate opening, 2 = gate closing) as the within-subject factors. We observed reaction time switch costs to be significantly determined by the *task variant* (*F*(1.58, 58.53) = 6.61, *p* = 0.005, η^2^_*p*_ = 0.05), the *gate switch mode* (*F*(1, 37) = 64.96, *p* < 0.001, η^2^_*p*_ = 0.31), and the *task variant∗gate switch mode* interaction (*F*(1.80, 66.57) = 7.40, *p* = 0.002, η^2^_*p*_ = 0.04). Pairwise Bonferroni-corrected t-tests suggested that in all task variants gate closing was associated with larger reaction time costs compared to gate opening (see Figure 3 part B) (purely visual task: (*t*(37) = 5.07, *p*_*corr*_ < 0.001, *d* = 1.34); auditory context cue task: *t*(37) = 9.14, *p*_*corr*_ < 0.001, *d* = 1.51; letter aurally: *t*(37) = 4.66, *p*_*corr*_ < 0.001, *d* = 1.06). Further, no between-task differences in reaction time switch costs were evident during gate opening (*F*(2, 74) = 0.15, *p*_*corr*_ = 0.1, BF = 0.09 ± 0.83%). Yet, reaction time switch costs during gate closing significantly differed across tasks (*F*(1.68, 62.08) = 13.39, *p*_*corr*_ < 0.001, η^2^_*p*_ = 0.12). Particularly, reaction time switch costs were higher in the auditory context cue task than the purely visual task (*t*(37) = 3.34, *p*_*corr*_ = 0.006, *d* = 0.26) and higher in the auditory context cue compared to the auditory item task (*t*(37) = 4.30, *p*_*corr*_ < 0.001, *d* = 0.44), while the purely visual and the auditory item task did not differ from each other (*t*(37) = 2.43, *p*_*corr*_ = 0.060).

Overall, this analysis incorporating reaction time outliers and participants excluded due to insufficient EEG data quality replicates the reported main findings. During gate opening, no differences between tasks were observed in either accuracy or reaction time measures. During gate closing, differences emerged only in reaction time, whereas accuracy did not differ between tasks. Specifically, reaction time costs were greatest in the auditory context cue task. This finding underscores that the sensory format of the context cue drives behavioral differences between tasks.

### Relationship between auditory pitch discrimination and reaction time switch costs during gate closing

To further account for the possibility that task difficulty in combination with individual differences in auditory processing ability could have driven the dissociation between the audiovisual task variants, we calculated the correlation between each participants’ z-transformed accuracy in the auditory frequency discrimination task and their z-transformed reaction time switch costs during gate closing. This analysis suggested no association between individual frequency discrimination accuracy and gate-closing-related reaction time switch costs in either the auditory context cue task (*r* = 0.16, *p* = 0.370) or the auditory item task (*r* = 0.00, *p* = 1), supporting the idea that task differentiation was not solely driven by differential task difficulty.

### Neurophysiological results

Given that behavioral results suggest that, when comparing audiovisual stimuli with purely visual stimuli, it is specifically gate closing that is modulated, the neurophysiological analyses focused on this gate switch mode by comparing switch/comparison trials (in which the gate is closing) with non-switch/comparison trials (in which the gate is already closed). The EEG trial rejection rates for both conditions can be found in Table 4. A detailed summary of the within-task sensor-level cluster statistics is provided in Table 5. For significant sensor-level contrasts, corresponding source activity profiles were estimated using DICS beamforming^33^ and subsequently summarized by applying the DBSCAN^34^ clustering algorithm. The resulting clusters provide a descriptive indication of the cortical regions demonstrating the largest modulation in the direction of the sensor-level contrast but were not subjected to statistical inference. For the estimation of the within-task contrasts, the DBSCAN was set to consider the top 1% voxels for clustering. The results of the within-task comparisons are illustrated in Figures 4, 5, and 6.

**Table 4:** EEG trial rejection rates for the conditions used to evaluate the gate closing effect in the three Reference Back variants

	purely visual	Context cue aurally	Letter aurally
switch/comparison	10.0 (8.1)	14.5 (9.3)	12.3 (8.8)
no-switch/comparison	10.6 (9.1)	15.9 (10.4)	12.2 (9.4)

Rejection rates in %. Rejection rates were calculated by dividing the number of retained trials by the number of trials per condition and subtracting this difference from 1. Given are the means with standard deviations in parentheses.

**Table 5:** Results of the within-task CBPT identifying gate closing-related activity modulations on sensor level

	Positive cluster (*p*-value)	Negative cluster (*p*-value)	Number of significant clusters
TBA			
Purely Visual	4 (*p* ≥ 0.138)	2 (*p* = 1.00)	0
Auditory Context Cue	1 (*p* = 0.054)	3 (*p* ≥ 0.719)	0
Letter Aurally	2 (*p* ≥ 0.635)	3 (*p* ≥ 0.010)	1
ABA			
Purely Visual	1 (*p* = 0.092)	1 (*p* = 0.008)	1
Auditory Context Cue	4 (*p* ≥ 0.691)	2 (*p* ≥ 0.002)	1
Letter Aurally	/	2 (*p* ≥ 0.002)	1
BBA			
Purely Visual	2 (*p* = 1.00)	4 (*p* ≥ 0.002)	1
Auditory Context Cue	4 (*p* = 1.00)	4 (*p* ≥ 0.002)	1
Letter Aurally	/	3 (*p* ≥ 0.002)	1

TBA = theta band activity, ABA = alpha band activity, BBA = beta band activity.

**Figure 4:**
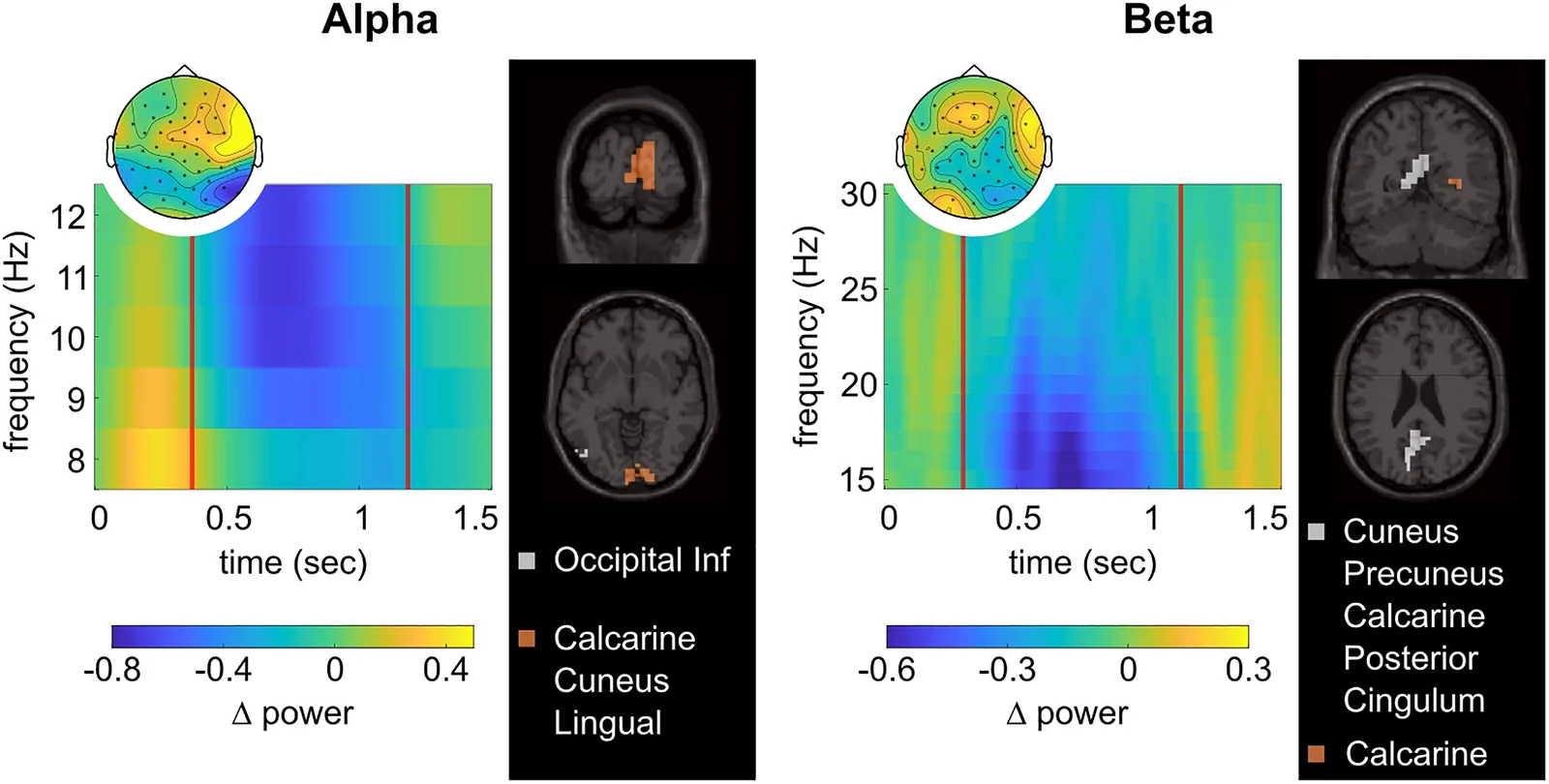
Neurophysiological modulations associated with gate closing within the purely visual task variant The left side of each subplot shows sensor-level results with time-frequency plots and topographic plots, visualizing the mean within-task power difference between switch/comparison trials and no-switch/comparison trials averaged across significant electrodes. The location of the significant electrodes in cluster-based permutation testing is indicated in the topographic plots. Time intervals exhibiting a significant gate-closing-related activity modulation are demarcated through red vertical boundaries in the time-frequency plots. Black boxes on the right side of each subplot contain the descriptive source-level results showing the anatomical localization of the top 1% activity modulations as identified by the DBSCAN for the gate-closing contrast. Different colors are used to mark independent clusters. Clusters are overlaid on the “Colin-27” brain anatomical template.^35^

**Figure 5:**
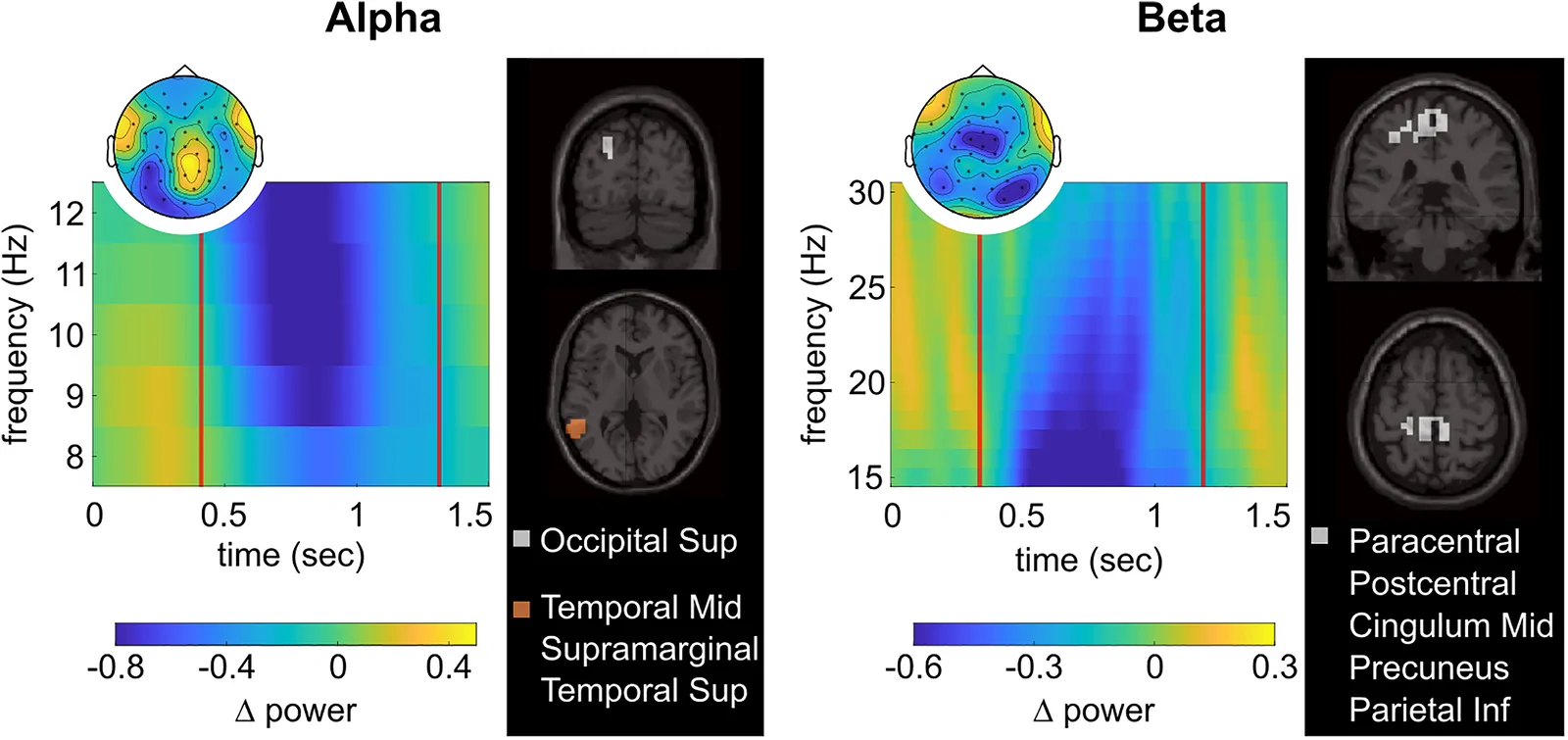
Neurophysiological modulations associated with gate closing within the auditory context cue task variant The left side of each subplot shows sensor level results with time-frequency plots and topographic plots, visualizing the mean within-task power difference between switch/comparison trials and no-switch/comparison trials averaged across significant electrodes. Location of the significant electrodes in cluster-based permutation testing is indicated in the topographic plots. Time intervals exhibiting a significant gate-closing-related activity modulation are demarcated through red vertical boundaries in the time-frequency plots. Black boxes on the right side of each subplot contain the descriptive source-level results showing the anatomical localization of the top 1% activity modulations as identified by the DBSCAN for the gate closing contrast. Different colors are used to mark independent clusters. Clusters are overlayed on the “Colin-27” brain anatomical template.^35^

**Figure 6:**
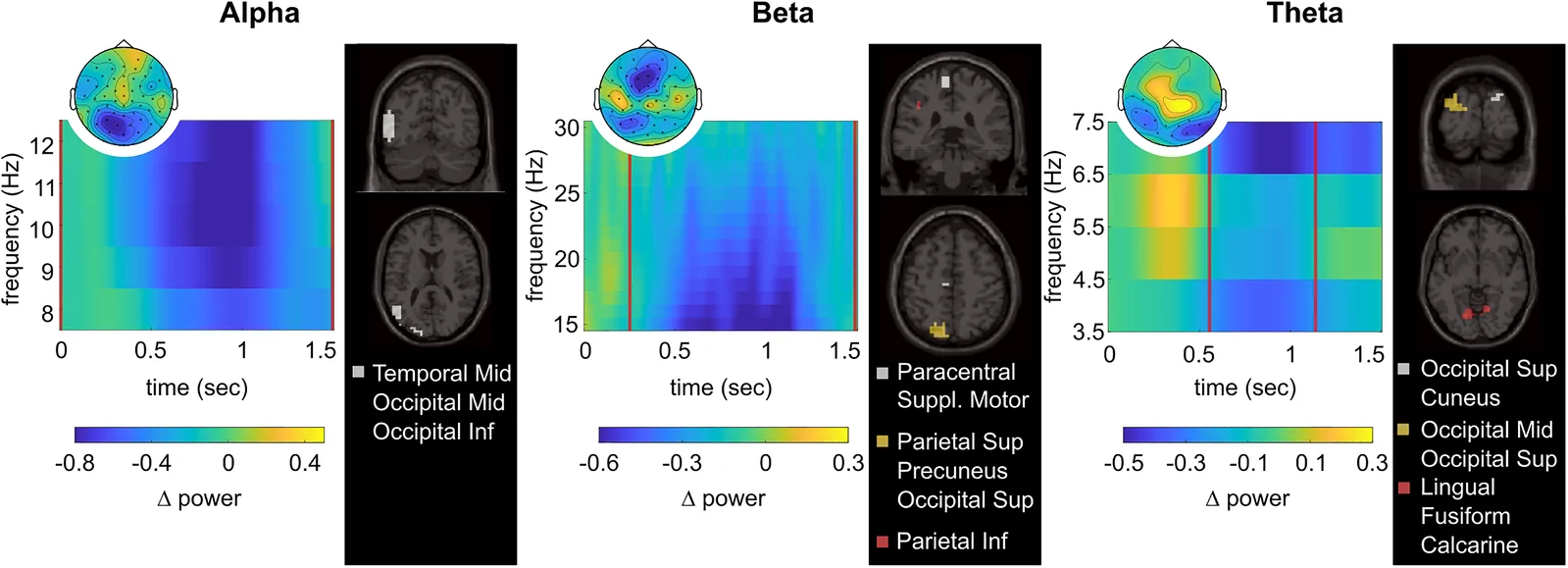
Neurophysiological modulations associated with gate closing within the auditory letter task variant The left side of each subplot shows sensor level results with time-frequency plots and topographic plots, visualizing the mean within-task power difference between switch/comparison trials and no-switch/comparison trials averaged across significant electrodes. Location of the significant electrodes in cluster-based permutation testing is indicated in the topographic plots. Time intervals exhibiting a significant gate-closing-related activity modulation are demarcated through red vertical boundaries in the time-frequency plots. Black boxes on the right side of each subplot contain the descriptive source-level results showing the anatomical localization of the top 1% activity modulations as identified by the DBSCAN for the gate closing contrast. Different colors are used to mark independent clusters. Clusters are overlayed on the “Colin-27” brain anatomical template.^35^

### Purely visual task variant

The within-task cluster-based permutation test (CBPT) contrasting switch/comparison and non-switch/comparison trials to identify activity modulations associated with gate closing (Figure 4) revealed no clusters of TBA modulation, a relative downmodulation of ABA during gate closing concentrated within a single cluster occurring between 367 ms and 1184 ms post stimulus onset (*T*_*sum*_ = −25,610.14, *p* = 0.008), as well as a single cluster of BBA downmodulation evident between 297 ms and 1211 ms after stimulus onset (*T*_*sum*_ = −25,749.26, *p* = 0.002).

The most pronounced ABA modulations during gate closing were associated with one cluster consisting of the bilateral calcarine cortices (BA 17), bilateral cuneus (BA 17), right lingual gyrus (BA 18), right superior occipital gyrus (BA 18/BA 19), and right inferior occipital gyrus (BA 18/BA 19). A second cluster was located in the left inferior occipital cortex (BA 18/BA 19). BBA modulations related to gate closing in the purely visual task were observed in one cluster constituted by the right calcarine sulcus (BA 17) and a second cluster encompassing the bilateral cuneus (BA 17) and precuneus (BA 7), the left calcarine sulcus (BA 17), bilateral posterior cingulum (BA 23/BA 31), and left superior occipital lobe (BA 18/BA 19).

### Task variant presenting the context cue aurally

In the task variant presenting the context cue aurally, no clusters of TBA modulations were obtained, but a downmodulation of ABA occurring between 410 ms and 1313 ms relative to stimulus onset (*T*_*sum*_ = −35,425.61, *p* = 0.002) and a downmodulation of BBA evident between 336 ms and 1184 ms post stimulus onset (*T*_*sum*_ = −35,544.00, *p* = 0.002) were observed (Figure 5).

The strongest ABA downmodulations associated with gate closing originated from two left-lateralized clusters: The first cluster spanning across the middle temporal gyrus (BA 21), supramarginal gyrus (BA 40), superior temporal gyrus (BA 22/BA 42), angular gyrus (BA 39), and inferior parietal cortex (BA 40), whereas the superior occipital (BA 18/BA 19) and middle occipital gyrus (BA 19) constituted the second cluster. In the auditory trial-type task, the bilateral paracentral lobule (BA 1–4), left precentral (BA 4) and postcentral gyrus (BA 1–3), left midcingulate cortex (BA 24/BA 32), bilateral precuneus (BA 7), left inferior parietal cortex (BA 39/BA 40), and right supplementary motor area (BA 6) formed a single cluster of greatest BBA modulations.

### Task variant presenting the letter aurally

In the task variant presenting the letter aurally, a single negative cluster in TBA was observable from 555 ms to 1137 ms after stimulus onset (*T*_*sum*_ *=* −7,559.98, *p* = 0.010) in addition to the downmodulation of ABA extending across the whole 1500 ms trial duration (*T*_*sum*_ = −55,459.23, *p* = 0.002) and a BBA downmodulation evident between 258 ms and 1500 ms relative to stimulus onset (*T*_*sum*_ = −52,282.34, *p* = 0.002; Figure 6).

The neuroanatomical structures linked to the greatest TBA downmodulations related to gate closing were distributed across three clusters: The first cluster contains the lingual gyrus bilaterally (BA 18), the right fusiform gyrus (BA 37), and the left calcarine cortex (BA 17); a second cluster comprises the left middle occipital gyrus (BA 19) and left superior occipital gyrus (BA 18/BA 19); and a third cluster comprises the right superior occipital gyrus (BA 18/BA 19) and the right cuneus (BA 17). A left-lateralization of greatest ABA downmodulations was observed, for which one cluster consisting of the middle temporal cortex (BA 21), middle occipital gyrus (BA 19), inferior occipital gyrus (BA 18/BA 19), superior occipital gyrus (BA 18/BA 19), inferior temporal gyrus (BA 20), and angular gyrus (BA 39) was observable. Lastly, the most pronounced BBA decreases during gate closing in the auditory letter task were distributed across three clusters. The first cluster was confined to the left inferior parietal lobule (BA 39/BA 40). The second, left-lateralized cluster spanned across the superior parietal cortex (BA 7), precuneus (BA 7), cuneus (BA 17), and superior occipital cortex (BA 18/BA 19). The third cluster incorporated the bilateral paracentral lobule (BA 1–4) and supplementary motor area (BA 6) as well as the left precuneus (BA 7).

### Comparison of task variants

Despite the qualitative differences in TBA modulation, no significant between-task differences in the magnitude of TBA modulations were evident for any task contrast (see Table 6 for a summary of the number of clusters observed; Figure 7 for an illustration of significant differences). Between-task differences in ABA were limited to the contrast of the purely visual and the auditory letter task variant, for which one positive cluster between 0 ms and 469 ms relative to stimulus onset emerged (*T*_*sum*_ = 5,275.16, *p* = 0.040). A distinction between the purely visual and the auditory letter task variant was also evident for BBA modulations in the time window between 836 ms and 1492 ms post-stimulus onset (*T*_*sum*_ = 9109.18, *p* = 0.002). Additionally, BBA modulations occurring between 1113 ms and 1473 ms after stimulus onset differed between the auditory context cue and auditory letter version (*T*_*sum*_ = 3,115.53, *p* = 0.016). The purely visual and auditory context cue task variants did not differ in the magnitude of gate-closing-related BBA modulations.

**Table 6:** Results of the between-task CBPT contrasting gate closing related activity modulations on sensor level

	Positive cluster (*p*-value)	Negative cluster (*p*-value)	Number of significant clusters
TBA			
PV - AT	2 (*p* ≥ 0.546)	/	0
PV - AL	4 (*p* ≥ 0.058)	1 (*p* = 1.00)	0
AT - AL	3 (*p* ≥ 0.058)	2 (*p* ≥ 0.855)	0
ABA			
PV - AT	3 (*p* ≥ 0.687)	/	0
PV - AL	6 (*p* ≥ 0.040)	/	1
AT - AL	8 (*p* ≥ 0.056)	/	0
BBA			
PV - AT	5 (*p* ≥ 0.086)	4 (*p* ≥ 0.713)	0
PV - AL	8 (*p* ≥ 0.002)	1 (*p* = 1.00)	1
AT - AL	16 (*p* ≥ 0.016)	1 (*p* ≥ 0.128)	1

TBA = theta band activity, ABA = alpha band activity, BBA = beta band activity, PV = purely visual task, AT = auditory context cue task, AL = auditory letter task.

**Figure 7:**
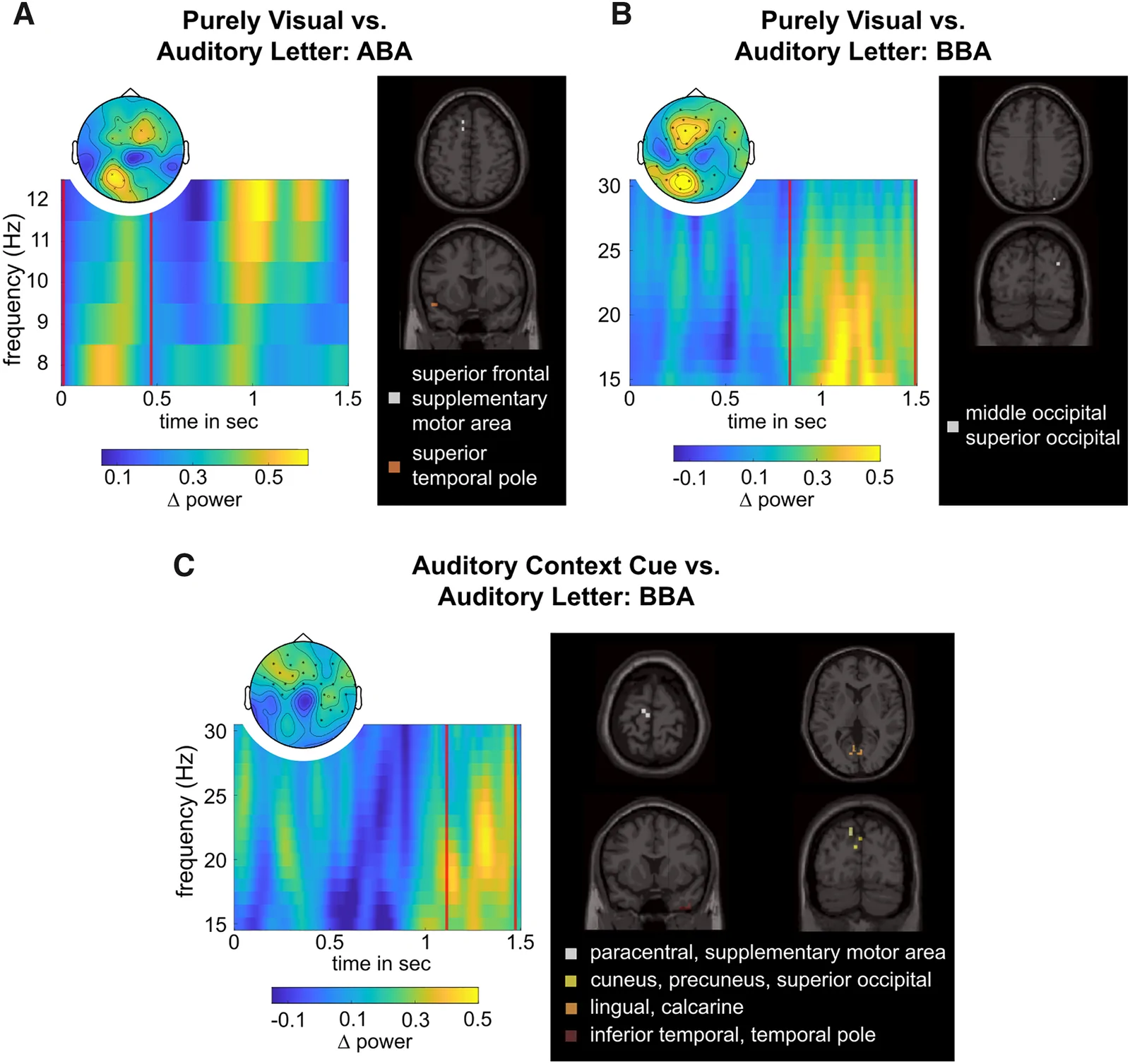
Differences in neurophysiological modulations associated with gate closing between task variants Figure parts (A) and (B) show the difference in gate closing associated neurophysiological modulations between the purely visual and auditory letter task (A: alpha band activity differences; B: beta band activity differences), whereas figure part (C) shows the difference between the auditory context cue and auditory letter task. The left side of each subplot shows sensor level results with time-frequency plots and topographic plots, visualizing the mean within-task power difference between switch/comparison trials and no-switch/comparison trials averaged across significant electrodes. Location of the significant electrodes in cluster-based permutation testing is indicated in the topographic plots. Time intervals exhibiting a significant gate-closing-related activity modulation are demarcated through red vertical boundaries in the time-frequency plots. Black boxes on the right side of each subplot contain the descriptive source-level results showing the anatomical localization of the top 10% activity modulations as identified by the DBSCAN for the gate closing contrast. Different colors are used to mark independent clusters. Clusters are overlayed on the “Colin-27” brain anatomical template.^35^

For the between-tasks comparison of significant frequency band modulations associated with gate closing, the DBSCAN was set to consider the top 10% voxels for clustering. The ABA modulation difference apparent for the contrast of the purely visual compared to auditory letter task was linked to one cluster encompassing the left superior temporal pole (BA 38) and one cluster constituted by the left supplementary motor area (BA 6), left superior frontal (BA 8–10) and medial superior frontal cortex (BA 8/BA 9). For the BBA modulation difference between the purely visual and auditory letter task, one right-lateralized cluster involving the middle occipital (BA 19) and superior occipital gyrus (BA 18/BA 19) was identified. Furthermore, the gate-closing-related BBA modulation disparity of the auditory context cue and auditory letter task was associated with seven distinct clusters. One cluster encompassed the left precuneus (BA 7) and superior occipital cortex (BA 18/BA 19); a second cluster contained voxels of the bilateral cuneus (BA 17). Cluster three involved the bilateral lingual gyrus (BA 18) and calcarine sulcus (BA 17), while in cluster four only the right-lateralized lingual gyrus (BA 18) and calcarine sulcus (BA 17) were implicated. Cluster five indicated the left paracentral lobule (BA 1–4) and supplementary motor area (BA 6). Cluster six contained the right insula (BA 13), right superior temporal pole (BA 38), and orbital part of the right inferior frontal gyrus (BA 45/BA 47). Lastly, cluster seven incorporated the right inferior temporal cortex (BA 20) and right middle temporal pole (BA 38).

## Discussion

Integrating prominent conceptualizations of WM in terms of interacting modality-specific sub-storages^8^^,^^9^ and the concept of working memory gating,^4^^,^^6^^,^^13^^,^^16^ we aimed to identify whether WM gating control operates on a supramodal basis or is subject to multimodal modulations. For this purpose, we recorded EEG signals while participants performed a purely visual and two audiovisual variants of the Reference Back task, allowing us to contrast behavioral performance indicators (i.e., switch costs) and neurophysiological signatures reflective of gating mechanisms.

On the behavioral level, we replicated the typical direction of switch cost effects reported in previous visual implementations of the task: accuracy decreases were greater for gate opening than gate closing, whereas RT costs were greater for gate closing than gate opening.^12^^,^^14^^,^^15^^,^^36^^,^^37^ While switch costs during gate opening were comparable across visual and audiovisual task variants, task-related differences emerged for the magnitude of RT costs, but not accuracy costs, during gate closing. This indicates that opening the WM gate to update representations and encode new information operates with comparable behavioral efficiency across the investigated modalities, whereas processes supporting the shielding and maintenance of working memory representations are influenced by the stimulus modality. Specifically, gate closing RT costs were lower in the purely visual and the auditory letter tasks compared to the auditory context cue task, suggesting that gating performance was not driven by a general distinction between multisensory versus unisensory task requirements. Instead, differences in gating performance seemed to be determined by the modality of the context cue, with superior performance when this cue was presented visually rather than aurally. One likely explanation is that tone pitch is a rather fragile stimulus feature that varies on a continuous scale and therefore requires constant articulatory rehearsal for an accurate representation.^38^ Particularly in the auditory context cue task, the need to compare the relative tone pitch across consecutive trials to determine the current trial type also necessitates a continuous updating of the tone pitch representation. This updating requirement is in conflict with the demand to close the WM gate in switch/comparison trials and thus might result in the increased gate-closing switch costs unique to the auditory context cue task. Together, these findings suggest that working memory gate opening and gate closing rely on dissociable control operations rather than sharing a common mechanistic basis that can adapt the WM gate in either direction.

To elucidate the neurophysiological mechanisms underlying modality-dependent costs during gate closing, we first characterized the oscillatory dynamics supporting gate closing within each task variant. In all task variants, gate closing was associated with decreased ABA and BBA in the switch compared to no-switch trials, consistent with previous work on purely visual tasks.^14^ As ABA is suggested to reflect selective attention, which is likely directed at the response-relevant item,^18^^,^^19^^,^^20^^,^^21^ the observed ABA reduction would indicate a relative disinhibition or broadening of attentional focus accompanying gate closing. On a conceptual level, this pattern could reflect a shift toward a less restrictive processing mode that enables the further integration of the context information that is relevant to inform the reconfiguration of the gating state. Importantly, source localization indicated that these processes are shaped by stimulus modality: in the purely visual task, ABA modulations were localized to visual cortical regions, whereas in the auditory trial-type task they further extended to supramodal association cortices (e.g., supramarginal and angular gyri) and temporal regions associated with auditory and semantic processing.^39^^,^^40^^,^^41^^,^^42^ A similar pattern emerged in the auditory letter task, suggesting that ABA primarily reflects modality-specific integration processes that vary according to the uni-versus multisensory nature of the task.

In contrast, BBA reductions likely reflect a release from the maintenance of the current cognitive state, consistent with the established role of BBA in preserving the “status quo.”^25^^,^^26^^,^^43^ Given this proposed role of BBA, the stronger BBA decrease during switch compared to no-switch trials would imply increased updating when the gate has to be closed. At first glance, this interpretation appears counterintuitive as comparison trials require maintaining the current item representation and, thus, the increased updating cannot be attributed to changes in WM content. However, what has to be updated is the state of the WM gate, as it has to be closed from a previously open state in switch trials. Although the present data do not directly establish this mechanism, one plausible interpretation is that the observed updating-related decrease in BBA may reflect the updating of the WM gate state instead of the WM content. With respect to the source estimation, the involved regions differed between the task variants. In the purely visual task variant, this modulation extended from basic visual processing regions into the retrosplenial cortex (i.e., precuneus and posterior cingulate cortex), which has been suggested to function as an association hub integrating sensory, long-term memory, and action-related information.^44^^,^^45^^,^^46^ Thus, the observed BBA modulations in this region during gate closing might reflect the updating of associations between sensory and action-related information within this integrative hub.^47^ This interpretation aligns with the literature on event file coding, in which the stability of event files (i.e., episodic memory traces containing an integrated representation of stimulus and response features) is related to BBA, with BBA desynchronizations enabling a reset of stimulus-response associations.^25^^,^^48^^,^^49^^,^^50^ Moreover, switching to gate closing in the auditory context cue task was accompanied by BBA downmodulations in bilateral sensory-motor regions (i.e., precentral and postcentral gyrus as well as the paracentral lobule) and the mid-cingulate cortex. The involvement of sensory-motor regions in conjunction with the mid-cingulate cortex, which has been linked to cognitive control functions,^51^^,^^52^ suggests a reconfiguration of the sensory-motor state under cognitive control. Given that the item-to-response-button assignment remains stable across consecutive comparison trials and only changes at gate closing, BBA downmodulations at this stage might support the establishment of updated stimulus-response mappings, or in other terms, the establishment of a new event file.^25^^,^^48^^,^^49^^,^^53^ In the auditory letter task, BBA modulation was linked to the superior parietal cortex, which has been reported to serve a critical function for the manipulation of information in WM.^54^ Considering the additional involvement of the precuneus, the updating process during gate closing likely targets visual aspects of the task (e.g., color information),^55^ while the concurrent involvement of the paracentral lobule and supplementary motor area suggests that the sensory-motor state (i.e., the stimulus-response mapping) is also updated, consistent with findings from the auditory context cue task. Interestingly, the regions identified in the auditory letter task partially overlap with those observed in the other task variants. While the involvement of the paracentral gyrus is evident in both audiovisual tasks, more posterior regions around the precuneus are implicated in both tasks in which the trial-type cue, and thus the indicator of the WM gate state, is presented visually. This suggests that while updating of sensory-motor information represents a general process during gate closing using audiovisual information, the updating of associations between (visual) sensory input, memory, and action is particularly pronounced when the requirement to change the WM gate state is conveyed visually rather than aurally. In this way, the neurophysiological findings align with the behavioral results, suggesting that modality-dependent differences in gate-closing performance may be driven by differences in how internal and external information are integrated and updated.

Analyses of TBA revealed that gate closing was associated with a downmodulation of posterior TBA only in the auditory letter task, whereas no significant effects were observed in the other task variants. Distributed TBA has repeatedly been linked to successful encoding and manipulation of WM contents,^31^^,^^56^^,^^57^ processes that are likely interrupted when the gate has to be closed. Consistent with this interpretation, TBA modulation was localized primarily to occipital regions, particularly the bilateral lingual gyri, which are implicated in color integration and visual-verbal processing.^58^^,^^59^ It might be speculated that this pattern suggests a selective disruption of ongoing encoding processes, which may be more pronounced when the item is presented aurally, as auditory input may be more intrusive and thus more likely to enter WM even when irrelevant.^60^ However, direct statistical comparisons between task variants did not reveal significant differences in TBA modulation, indicating that these effects remain subtle.

In conjunction, the within-task evaluations revealed distinct computational roles of the investigated frequency bands during WM gate closing. Specifically, ABA downmodulations appear to effectuate a more integrative processing mode that might support the establishment of a link between the presented item and the context-cue-mediated task rule. Adjusting the gate state according to the cued task rule is then governed by BBA modulations and involves a reconfiguration of sensory-motor representations. TBA modulations, however, were not consistently observed across tasks, suggesting that TBA is not a robust neurophysiological indicator of gate closing processes, likely due to its more prominent association with WM encoding and updating.^31^^,^^56^^,^^57^

Despite ABA and BBA showing similar functional roles during gate closing in all task variants, a dissociation between tasks was evident with regard to the relative magnitude of these modulations. In particular, robust differences emerged between the auditory letter task and the other task variants, suggesting that neurophysiological differences are primarily driven by the modality of the item. In the purely visual task, early relative ABA upregulation in switch-to-gate-closing trials was more pronounced than in the auditory letter task. This effect was mainly driven by lower ABA power in consecutive no-switch trials in the visual task compared to the auditory letter task, whereas ABA levels during switch trials were comparable across tasks. Source localization linked this difference to medial frontal regions and the superior temporal pole, regions associated with verbal WM and auditory speech processing,^61^^,^^62^ with the temporal pole additionally functioning as a multimodal semantic hub.^63^^,^^64^^,^^65^^,^^66^ The reduced suppression of these phonologically relevant regions in the purely visual task suggests a transformation of visually presented letters into a phonological code, whereas in the auditory letter task this transformation is unnecessary, allowing for more efficient suppression of irrelevant representations. Given that this effect is most pronounced during repeated comparison trials, it likely reflects differences in the format of comparison between current and stored items rather than differences in WM storage per se. In BBA, both task variants presenting the item visually differed from the auditory letter task in the post-response time window. While BBA desynchronization accompanied gate closing in all task variants, its magnitude was greatest in the auditory letter task. Moreover, absolute BBA levels across both switch and no-switch trials were lower in the auditory letter task than in the other task variants, suggesting a reduced maintenance tendency even when shielding of WM contents would be required. This pattern aligns with the hypothesis that auditory stimuli, due to their dynamic and transient nature, induce a stronger tendency toward updating. The spatial distribution of these BBA differences was primarily confined to occipital regions when contrasting visual and auditory item presentation, while additional involvement of extrastriate visual, sensory-motor, and inferior frontal and temporal regions points to differences in the integration and control of task-relevant information.^67^ Notably, the largest differences across a broad set of regions emerged between the two audiovisual task variants, whereas differences between visual and auditory item presentation were mainly restricted to visual sensory regions. This pattern suggests that modality effects on working memory gating do not arise from isolated sensory channels but from the interaction between item and contextual modalities.

### Limitations of the study

The current study provides a comprehensive neurobehavioral account of how WM gating mechanisms are shaped by different sensory constraints, highlighting the importance of widespread integration processes between sensory channels. However, to attain a deeper understanding of the precise mechanisms underlying this integration, subsequent research should be dedicated to determine whether communication between sensory channels follows a hierarchical organization, for example, where visual attention to context cues facilitates the processing of an auditory item, or vice versa. To address these issues, future studies could utilize analytic approaches such as phase-amplitude coupling^68^^,^^69^^,^^70^ or dynamic causal modeling.^71^^,^^72^^,^^73^

A further specific limitation is linked to the conceptualization of our task variants. Compared to the categorical discrimination of different colors, the comparison of relative tone pitches required to discern the current context cue might have been more taxing. This is likely because tone pitch varies along a more continuous scale and is rather fragile, thereby creating a greater necessity not only for continuous rehearsal but also for constant updating of the tone pitch representation. This increased demand may have contributed to the overall lower accuracy during the auditory context cue task compared to the task variants presenting the context cue visually. However, task difficulty alone is unlikely to be the primary driver of our observed task differences. First, it does not readily explain why behavioral differences were confined to the gate closing condition only. Moreover, if the observed gate closing effects primarily reflected the increased demands associated with auditory pitch discrimination, individual differences in pitch discrimination performance should have been associated with gate closing switch costs. However, no such relationship was observed. Second, on the neurophysiological level, we would have expected differences in task difficulty to be mirrored by enhanced frontal-midline TBA signaling heightened cognitive demand^32^ or increased alpha-band desynchronization reflecting broader attentional deployment.^19^^,^^20^ Yet the most pronounced between-task differences emerged in BBA instead. Nevertheless, to fully rule out any confounding effects of task difficulty, a suitable alternative to the use of tone pitches would be different sound categories that should not easily evoke an associated visual image.

The generalizability of our findings might also be constrained by the composition of our sample, with a sex ratio skewed toward a relative predominance of female participants. As our study was not powered to investigate sex-related differences, the results may not fully capture sex-specific nuances.

These considerations primarily concern the extent to which the present findings can be generalized beyond the specific task and sample investigated here. At the level of interpretation of the neurophysiological results, however, some additional caution is warranted. As our source estimates partially linked the observed BBA effects to regions traditionally associated with the motor system, a contribution of motor-related processes cannot be fully excluded. However, the predominantly post-response timing of the effects and their distributed source localization extending beyond classical motor regions argue against motor processes as the major driver of the observed BBA modulations.

### Conclusions

Our findings show that working memory gating is not controlled by a unitary, amodal mechanism, but is shaped by the sensory structure of task-relevant information. Performance during gate opening, which enables updating, appears to be largely independent of modality. In contrast, gate closing, which supports stabilization and shielding of working memory contents, depends on how relevant information is distributed across sensory modalities. This dissociation points to two functionally distinct control regimes: a supramodal process for flexible updating and a modality-dependent process for maintaining and protecting internal representations. These results link modular accounts of working memory architecture with gating models. The neural findings suggest that oscillatory rhythms implement distinct computational functions: ABA supports the integration of task-relevant information, whereas BBA contributes to reconfiguring the gate state. The findings indicate that control over working memory must be understood in relation to multisensory environments. Modality effects in gating emerge from interactions between item and contextual modalities, showing that the organization of sensory information shapes how access to working memory is regulated.

## Resource availability

### Lead contact

Requests for further information and resources should be directed to and will be fulfilled by the lead contact, Astrid Prochnow (astrid.prochnow@ukdd.de).

### Materials availability

This study did not generate new unique reagents.

### Data and code availability

• Behavioral and EEG raw data have been deposited at Zenodo.org (https://doi.org/10.5281/zenodo.22031920) and are publicly available as of the date of publication.
• All original code has been deposited at Zenodo.org (https://doi.org/10.5281/zenodo.22031920) and is publicly available as of the date of publication.
• Any additional information required to reanalyze the data reported in this paper is available from the lead contact upon request.

## Acknowledgments

We thank all participants taking part in the study. This work was supported by a grant from the 10.13039/501100003042Else Kröner-Fresenius-Stiftung to A.P. (2024_EKSE.104).

## Author contributions

RL: conceptualization, methodology, software, formal analysis, investigation, data curation, writing—original draft, visualization; CB: conceptualization, methodology, resources, writing—review and editing, supervision, project administration, funding acquisition; AP: conceptualization, methodology, writing—review and editing, visualization, supervision, project administration, funding acquisition.

## Declaration of interests

The authors declare no competing interests.

## STAR★Methods

### Key resources table

**Table:** 

REAGENT or RESOURCE	SOURCE	IDENTIFIER
**Deposited data**		

Raw data behavior	This paper	https://doi.org/10.5281/zenodo.22031920
Raw data EEG	This paper	https://doi.org/10.5281/zenodo.22031920
Custom Code	This paper	https://doi.org/10.5281/zenodo.22031920

**Software and algorithms**		

MATLAB	https://de.mathworks.com/products/matlab.html	RRID:SCR_001622
BrainVision Recorder	https://www.brainproducts.com/productdetails.php?id=21	RRID:SCR_016331
BrainVision Analyzer	https://www.brainproducts.com/solutions/analyzer/	RRID: SCR_002356
EEGLAB	https://github.com/sccn/eeglab	RRID:SCR_007292
FieldTrip	https://www.fieldtriptoolbox.org	RRID:SCR_004849
ICLabel	https://github.com/sccn/ICLabel	RRID:SCR_016879
Presentation	http://www.neurobs.com/	RRID:SCR_002521
free text to speech generator	https://ttsfree.com/	N/A
Audacity	https://www.audacityteam.org/	RRID:SCR_007198
R	https://www.r-project.org/	RRID:SCR_001905

### Experimental model and study participant details

#### Participants

*N* = 41 adult human participants initially participated in the study. Among them, *N* = 1 participant did not complete all tasks, *N* = 1 participant presented with impaired hearing (see test of hearing acuity section). Furthermore, *N* = 1 participants was excluded due to high error levels (less than 60% accuracy across all three tasks) and *N* = 3 participants were determined as RT outliers (*N* = 1 per task; see section on computation of gating indices). Additionally, during EEG preprocessing *N* = 1 participant was excluded as more than a third of their channels required interpolation after bad channels were removed (see section on EEG recording and preprocessing for details) Consequently, *N* = 34 participants (25 female, 9 male according to sex assigned at birth) in an age range between 19 and 30 years (M = 24.3 years, SD = 3.0) were included in the data analysis. This final sample size is comparable to previous behavioral and neurophysiological studies using the “Reference Back” paradigm.^15^^,^^16^^,^^36^^,^^37^ Participants were recruited through an in-house database or through advertisements, and received course credit or a reimbursement of 10 EUR/h for their participation. All participants were right-handed, had normal or corrected-to-normal vision, no dyslexia and no current neuropsychological disorders. Informed consent was obtained from all participants before any study procedure was applied. Experiments were conducted in strict adherence to the institutional guidelines. The study was approved by the local ethics Committee of the Medical Faculty of TU Dresden (IRB00001473, approval number SR + BO-EK-523112021, date of approval September 17, 2024).

### Method details

#### Test of hearing acuity

To ensure hearing acuity of our participants, we conducted a pure tone audiometry using the automatic Hughson Westlake test of the MAICO MA33 audiometer (MAICO Diagnostics GmbH; Berlin, Germany) with the included noise-enclosure headset (type: DD65 v2). Hearing thresholds for each ear were obtained at 250Hz, 500Hz, 1 kHz, 2 kHz and 3kHZ. Hearing levels for both ears in all but three participants were within a normal range of below or equal to 25 dB. For two participants a one-sided mildly impaired hearing of 30 dB was recorded within a single frequency, while one-person presented a moderately severe hearing impairment of 60 dB for one frequency. As the two-participants with hearing levels of 30 dB showed no impairments in a frequency discrimination task (see below), we deemed their hearing levels to be sufficient to perform the tasks and thus did not exclude them.

Furthermore, participants’ ability to discriminate between frequencies was tested. For this frequency discrimination task we generated 39 pure tones evenly spaced between 250Hz and 1200Hz (25Hz increment between successive tones) using the *tone_generator* function^74^ for MATLAB R2022a (The Mathworks company; RRID:SCR_001622). The tones were then arranged in two vectors: the first one in ascending order from 250Hz to 1200Hz, the second one in descending order form 1200Hz–250Hz. Tones with the same position in both vectors were paired, which resulted in 38 pairs of tones that differed in their frequency and 1 pair of tones with the same frequency. To avoid participants expecting different only trials, we added 10 same tone pairs of randomly chosen frequencies. Lastly, we randomized the order in which the above described 49 tone pairs (38 different, 11 same) were presented. The trial sequence of the frequency discrimination task was as follows: Each trial started with a black fixation cross on white background being shown while two tones were presented over stereo headphones for 500 ms each, separated by a 750 ms break in between the tones. Afterward, a response prompt appeared instructing participants to indicate whether the two played tones were of a different frequency or the same frequency by pressing the left or right CTRL key with their index fingers, respectively. Only after a response was given, the next trial started, following an inter-trial interval of 1000 ms. The task was implemented in the software Presentation (version 20.0 09.04.17; RRID:SCR_002521).

#### Tasks

We exchanged the letters “X” and “O” that are used in the classical Reference Back task with the letters “A” and “U” to ensure that in the version with auditory presentation of letters performance differences do not emerge due to different discrimination speeds for spoken consonants compared to vowels. For the task implementation and recording of responses, the software Presentation (version 20.0 09.04.17) was used. Participants performed the tasks on a 19-inch computer monitor with a black background. Each task lasted approximately 30 min and was divided into 6 blocks with 120 trials each (720 trials per task), interleaved by breaks of self-adjustable length. All tasks were preceded by an exercise to get participants accustomed to the task. During the exercise, participants received continuous feedback (‘correct’ or ‘wrong’) on their performance and were only allowed to proceed to the main task after an above chance performance level was attained. The order in which participants completed the task variants was counterbalanced across participants. The different task variants are illustrated in Figure 1.

Each trial of the purely visual task variant started with a white fixation cross being presented in the center of the screen for 800 ms, before a white target letter (“U” or “A”) surrounded by either a blue frame (comparison trial) or red frame (reference trial) appeared for a maximum duration of 1500 ms. Within this time span, participants had to decide whether the current letter was different (left CTRL key) or the same (right CTRL key) as the last letter presented within a red frame. The combination of letter and frame disappeared as soon as a response was given or after a maximum duration of 1500 ms without a response. Afterward a blank screen was shown for 1000 ms before the next trial ensued. The task always started with a reference trial (red frame) that required no response and that was not included in the data analysis as no reference allowing the performance of a matching judgment is defined at this point. In following trials, the trial type (comparison or reference) and the shown letter stimulus were randomly selected while ensuring a balanced number of “different” and “same” responses. Furthermore, it was determined that in 30% of all trials the current trial type differs from the preceding trial (switch trials), whereas in the remaining 70% the trial type was constant across successive trials (no-switch trials).

To create the variant of the Reference Back task with the trial type being indicated aurally, we swapped the colored frames indicating the trial type for two pure tones of different frequencies that were created with a *tone_generator* function^74^ for MATLAB R2022A and presented over stereo headphones. A lower tone (500 Hz) indicated a comparison trial, while a higher tone (1200 Hz) indicated a reference trial. Consequently, participants were required to decide whether the letter that is currently presented on the screen is the same or different from the last latter that was accompanied by a high tone.

For the task variant with the letter being presented aurally, we kept the colored frames but replaced the written target letters with spoken letters. The letter audio was created using a free text to speech generator (https://ttsfree.com; selected language: German; Speaker: Conrad, default voice pitch and voice speed). The software Audacity (RRID:SCR_007198) was used to adjust the audio duration of spoken letters in order to match the 1500 ms maximum presentation duration of written letters. Instructions for this task variant read ‘Please indicate whether the letter you are currently hearing is different from or the same as the last letter presented concurrently with a red frame’.

#### Computation of gating indices

We based the main behavioral investigation of gating dynamics on the switch costs parameter, which reflects increases in reaction times (RT) and reduced accuracy (ACC) occurring when a switch in the gate state is required.^2^^,^^7^ For the computation of switch costs, we followed the analysis strategy described in Rac-Lubashevsky & Kessler^13^ by first calculating the mean ACC and mean correct RT per person and task for the conditions that result from a distinction of reference vs. comparison trials, switch vs. no-switch trials and match (‘same’ response) vs. mismatch (‘different’ response) trials. Switch costs for gate opening (i.e., switching from comparison to reference trials) were calculated by taking the difference in RT and ACC between switch and no-switch conditions in reference trials. Switch costs for gate closing (i.e., switching from reference to comparison trials) were computed based on the difference in RT and ACC between switch and no-switch conditions in comparison trials.

Before computing the gating indices, we preprocessed the behavioral data by removing all participants with lower than 60% mean ACC within a task. To exclude premature responses, we applied an RT filter by omitting all trials in which responses were given less than 100 ms after target stimulus onset. In Table 2 we provide an overview on the percentage of trials that were rejected due to being classified as premature or false responses. Furthermore, participants were determined as RT outliers when deviating more than 3 SD from the log10 transformed median RT switch costs in gate opening and closing conditions.

#### EEG recording and preprocessing

Continuous EEG recordings were obtained using a BrainAmp amplifier in combination with Brain Vision Recorder 1.2 software (Brain Products, Gilching, Germany; RRID: SCR_016331) and elastic EEG caps (Easycap, Wörthsee, Germany) equipped with 60 built-in Ag/AgCl electrodes arranged in an equidistant layout (reference electrode at FPz; ground electrode at θ = 58, φ = 78). Data were initially sampled at 500 Hz and later downsampled offline to 256 Hz. Preprocessing was carried out in MATLAB R2022a (The MathWorks Corp.) using the ‘Automagic’ pipeline^75^ together with EEGLAB (RRID: SCR_007292).^76^ First, 50 Hz line noise was removed via a multitaper algorithm implemented in PREP,^77^ after which data was re-referenced using a robust average reference computed from all good channels. To detrend the EEG data, a 0.5 Hz FIR high-pass filter (order 1286; stop-band attenuation 80 dB; transition band 0.25–0.75 Hz) included in EEGLAB’s clean_rawdata() function was applied. This function also identifies and removes bad channels exhibiting flatlines for more than 5 s, having low correlation with other channels, or displaying excessive temporal variability (standard deviation >25 μV). Additionally, clean_rawdata() detects time segments with strong noise bursts and reconstructs them using the Artifact Subspace Reconstruction (ASR; burst criterion: 15) method.^78^ This method evaluates the EEG data in short sliding time windows and identifies signal subspaces whose covariance structure deviates from that of clean reference segments. Artefact-contaminated subspaces are subsequently reconstructed based on the covariance structure of the clean data. All components that cannot be reconstructed are discarded. Subsequently, data were filtered using a 40 Hz low-pass filter (sinc FIR filter; order 86).^79^ Further artifact reduction involved applying the subtraction method described by Parra et al.^80^ to isolate EOG-related artifacts, followed by the Multiple Artifact Rejection Algorithm (MARA),^81^^,^^82^ which performs independent component analysis and removes components identified as muscle or eye-movement artifacts. For the identification and removal of components reflecting cardiac artifacts ICLabel (RRID: SCR_016879)^83^ was used. Finally, any missing or eliminated channels were reconstructed using a spherical interpolation method.

In a next step, the continuous EEG data were segmented into single trials using Brain Vision Analyzer version 2.1 (Brain Products, Inc.; RRID: SCR_002356). We set the length of each trial to 4000 ms, including 1000 ms before to 3000 ms after target stimulus onset, to avoid edge effects during the later time-frequency decomposition. To each trial, an automatic artifact rejection was applied to remove any remaining artifacts characterized by the following criteria: more than 150 μV difference within 200 ms, amplitude of < −100 μV or >100 μV or an activity of <0.5 μV for 200 ms. Trials free of artifacts and with correct responses were separated into the eight conditions as outlined in the section describing the computation of gating indices. The resulting percentage of excluded trials for the conditions constituting the gate closing contrast are summarized in Table 4.

#### Time-frequency decomposition of clusters

We subsequently performed a time-frequency analysis on the segmented EEG data using the FieldTrip toolbox (RRID: SCR_004849).^84^ Through application of a Morlet wavelet (length of 3 SD of the implicit Gaussian kernel, frequency range: 2-30Hz, number of cycles increasing linearly from 3 to 12) we decomposed the EEG signal into theta (4–7 Hz), alpha (8–12 HZ) and beta (15–30 Hz) band activity. Time-frequency representations of each frequency band were baseline-corrected (selected baseline: −200 ms–0 ms relative to target stimulus onset) by means of a decibel conversion as described in Yu et al.^36^ Given that we had observed greatest behavioral task differences for gate closing, we then determined which frequency bands exhibited activity modulations related to gate closing. This was achieved by performing a CBPT (number of permutations: 1000, *p*-value corrected at cluster level) that contrasted average power estimates in switch/comparison trials and no-switch/comparison trials within each task. This CBPT was applied to the time window from stimulus onset until 1500 ms after stimulus onset. To retrieve information about the timing of oscillatory activity differences between switch and no-switch trials, we set the CBPT to not average over time.

For all frequency bands showing modulations related to gate closing, we then compared the power difference between switch and no-switch/comparison trials in a between-task CBPT (number of permutations: 1000, *p*-value corrected at cluster level, no averaging of time) to examine task-specific differences in activity modulations related to gate closing.

All CBPTs outlined above were planned *a priori* to evaluate distinct hypotheses regarding oscillatory modulations within each frequency band and task. Accordingly, each CBPT was treated as a separate confirmatory analysis and therefore no additional correction for multiple comparisons across tests was applied.

#### Source reconstruction and beamforming

To identify the neuroanatomical sources of gate-closing-related activity modulations we first performed source reconstruction using the dynamic imaging of coherent sources (DICS) beamformer.^33^ For each frequency band that exhibited significant modulations associated with gate closing, we separately reconstructed the source level activity profiles for switch/comparison and no-switch/comparison trials within the significant time points identified in the within task CBPT. Within the DICS beamformer, a Hanning taper with a smoothing box of 0.5 Hz is applied to the frequencies of interest, in order to obtain a cross-spectral density matrix, which is then used to create a spatial filter for each participant. Based on the boundary element volume conduction model implemented in FieldTrip,^84^ activity is subsequently mapped onto a three-dimensional brain model defined in Montreal Neurological Institute (MNI) space, which is discretized into a grid with 0.5 cm spacing. Subsequently, we entered the average within task source level activity difference between switch and no-switch/comparison trials occurring within the significant time points, as identified in the within task CBPT, into the Density Based Spatial Clustering of Applications with Noise (DBSCAN).^34^ The DBSCAN was set to identify clusters of adjacent voxels showing the top 1% activity difference between conditions. Clusters had to consist of at least 5 voxels arranged within a search radius of 0.75 cm (epsilon = 1.5∗gridsize). Voxels within the cerebellum were disregarded. Assignment of identified voxels to neuroanatomical structures was achieved by applying the Automatic Anatomical Labeling (AAL) atlas,^85^ and the obtained clusters were visualized on the Colin-27 standard MRI anatomical template.^35^

Subsequently, we repeated the steps outlined above to identify the neuroanatomical structures associated with between-task differences in gate-closing-related activity modulations. To this end, we first reconstructed the source level activity profiles for switch/comparison and no-switch/comparison trials within the relevant frequency bands and significant time windows identified by the between task CBPT, using the DICS beamformer.^33^ Next, we derived the gate closing source activity profile for each task by calculating the difference between switch/comparison and no-switch/comparison trials, and then contrasted this activity difference between tasks. The resulting between-task source-level activity difference reflecting gate closing processes were subsequently entered into the DBSCAN^34^ to identify clusters of neighboring voxels displaying the greatest between-task differences. Apart from adjusting the threshold to 10%, all other parameters were kept identical to the within-task approach. Finally, the identified voxels were labeled using the AAL atlas.^85^

### Quantification and statistical analysis

Statistical analyses were conducted in R version 4.4.1 (R Core Team; RRID:SCR_001905). To evaluate how the within-subject factors *task variant* (1 = purely visual, 2 = context cue aurally, 3 = letter aurally), *trial type* (1 = reference trial, 2 = comparison trial) and *switching* (1 = switch, 2 = no-switch) affect task performance, we fitted a repeated measures ANOVA with subject IDs as the between subject factor, using the *anova_test()* function as provided in the rstatix package.^86^ Degrees of freedom for the within-subject factors were corrected using the Greenhouse-Geisser method in case of violated sphericity. Models were fitted separately with RT measures and accuracy measures as the dependent variable. In case of significant interactions, pairwise Bonferroni corrected t-tests were computed using the *pairwise_t_test*() function.

To analyze whether switch costs vary depending on the gate switch mode and task version, we further fitted a repeated measures ANOVA using the *anova_test()* function with the *gate switch mode* (1 = gate opening; 2 = gate closing) and the *task variant* (1 = purely visual, 2 = context cue aurally, 3 = letter aurally) as within-subject factors and subjects as a between-subject factor. Degrees of freedom for the within-subject factors were corrected using the Greenhouse-Geisser method when necessary. This model was fitted separately for ACC and RT based switch costs as the dependent variable. For ACC and RT measures separately, we also computed the difference between switch costs observed during gate opening and closing (gate opening minus gate closing) and conducted a repeated measures ANOVA to evaluate whether the extent of this switch cost difference between gate switch modes significantly differed between tasks.

In the case of null effects for between-task differences in gate opening and gate closing, we calculated Bayes Factors using the BayesFactor package in R^87^ to further evaluate the likelihood of the H0.

The behavioral analyses were conducted with the final sample of *N* = 34 as well as with *N* = 38 to examine the influence of excluded participants.

For all statistical tests, significance was defined as *p* < 0.05. Descriptive statistics are reported with mean and standard deviation from the mean. The exact statistical details are reported in the Results section. In the behavioral figures, statistical significance levels are illustrated with asterisks, as described in the respective figure legends (∗*p* < 0.050, ∗∗*p* < 0.010, ∗∗∗*p* < 0.001).
